# Supplementary material for: Study on Chemical Constituents of Panax notoginseng Leaves
Source: Molecules. 2023 Feb 27;28(5):2194. doi: 10.3390/molecules28052194 (PMC10004258; doi:10.3390/molecules28052194)
Supplement: Supplementary file 1 [file molecules-28-02194-s001.zip › molecules-2216549-supplementary.pdf]

SUPPLEMENTARY MATERIAL

Study on Chemical Constituents of *Panax notoginseng* Leaves

Xiao-Juan Sun[1], Hong-Bo Deng[1], Teng-Yun Shu[1], Li-Hua Su[1], Min Xu[1], Hai-Zhou Li[1,*]

[1] Center for Pharmaceutical Sciences, Faculty of Life Science and Technology, Kunming University of Science and Technology, Chenggong Campus, Kunming 650500, P. R. China

[*] Correspondence: lihaizhou@kust.edu.cn

Tel: 86-871-65920253

	Content	Page
Table S1.	^{1}H and ^{13}C NMR spectral data of **1**	6
Table S2.	^{1}H and ^{13}C NMR spectral data of **2**	7
Table S3.	^{1}H and ^{13}C NMR spectral data of **3**	8
Table S4.	^{1}H and ^{13}C NMR spectral data of **4**	9
Table S5.	^{1}H and ^{13}C NMR spectral data of **5**	10
Table S6.	^{1}H and ^{13}C NMR spectral data of **6**	11
Table S7.	^{1}H and ^{13}C NMR spectral data of **7**	12
Table S8.	^{1}H and ^{13}C NMR spectral data of **8**	13
Figure S1.	HMBC correlations of **1** (C_5D_5N)	1
Figure S2.	HMBC correlations of **2** (C_5D_5N)	2
Figure S3.	HMBC correlations of **3** (C_5D_5N)	2
Figure S4.	HMBC correlations of **4** (C_5D_5N)	3
Figure S5.	HMBC correlations of **5** (C_5D_5N)	3
Figure S6.	HMBC correlations of **6** (C_5D_5N)	4
Figure S7.	HMBC correlations of 7 (C_5D_5N)	5
Figure S8.	HMBC correlations of **8** (C_5D_5N)	5
Figure S9.	IR spectrum of **1**	14
Figure S10.	UV spectrum of **1**	14
Figure S11.	HRESI spectrum of **1**	15
Figure S12.	$[\alpha]^{23}_{D}$ of **1**	15
Figure S13.	^{1}H NMR spectrum of **1** in C_5D_5N at 298K (600 MHz)	16
Figure S14.	^{13}C NMR spectrum of **1** in C_5D_5N at 298K (150 MHz)	16
Figure S15.	HSQC spectrum of **1** in C_5D_5N at 298K	17
Figure S16.	HMBC spectrum of **1** in C_5D_5N at 298K	17
Figure S17.	IR spectrum of **2**	18
Figure S18.	UV spectrum of **2**	18
Figure S19.	HRESI spectrum of **2**	19
Figure S20.	$[\alpha]^{23}_{D}$ of **2**	19
Figure S21.	^{1}H NMR spectrum of **2** in C_5D_5N at 298K (600 MHz)	20
Figure S22.	^{13}C NMR spectrum of **2** in C_5D_5N at 298K (150 MHz)	20
Figure S23.	HSQC spectrum of **2** in C_5D_5N at 298K	21
Figure S24.	HMBC spectrum of **2** in C_5D_5N at 298K	21
Figure S25.	IR spectrum of **3**	22
Figure S26.	UV spectrum of **3**	22
Figure S27.	HRESI spectrum of **3**	23
Figure S28.	$[\alpha]^{23}_{D}$ of **3**	23
Figure S29.	^{1}H NMR spectrum of **3** in C_5D_5N at 298K (600 MHz)	24
Figure S30.	^{13}C NMR spectrum of **3** in C_5D_5N at 298K (150 MHz)	24
Figure S31.	HSQC spectrum of **3** in C_5D_5N at 298K	25
Figure S32.	HMBC spectrum of **3** in C_5D_5N at 298K	25

	Content	Page
Figure S33.	IR spectrum of **4**	26
Figure S34.	UV spectrum of **4**	26
Figure S35.	HRESI spectrum of **4**	27
Figure S36.	$[\alpha]^{23}_{D}$ of **4**	27
Figure S37.	^{1}H NMR spectrum of **4** in C_5D_5N at 298K (600 MHz)	28
Figure S38.	^{13}C NMR spectrum of **4** in C_5D_5N at 298K (150 MHz)	28
Figure S39.	HSQC spectrum of **4** in C_5D_5N at 298K	29
Figure S40.	HMBC spectrum of **4** in C_5D_5N at 298K	29
Figure S41.	IR spectrum of **5**	30
Figure S42.	UV spectrum of **5**	30
Figure S43.	HRESI spectrum of **5**	31
Figure S44.	$[\alpha]^{23}_{D}$ of **5**	31
Figure S45.	^{1}H NMR spectrum of **5** in C_5D_5N at 298K (600 MHz)	32
Figure S46.	^{13}C NMR spectrum of **5** in C_5D_5N at 298K (150 MHz)	32
Figure S47.	HSQC spectrum of **5** in C_5D_5N at 298K	33
Figure S48.	HMBC spectrum of **5** in C_5D_5N at 298K	33
Figure S49.	IR spectrum of **6**	34
Figure S50.	UV spectrum of **6**	34
Figure S51.	HRESI spectrum of **6**	35
Figure S52.	$[\alpha]^{23}_{D}$ of **6**	35
Figure S53.	^{1}H NMR spectrum of **6** in C_5D_5N at 298K (600 MHz)	36
Figure S54.	^{13}C NMR spectrum of **6** in C_5D_5N at 298K (150 MHz)	36
Figure S55.	HSQC spectrum of **6** in C_5D_5N at 298K	37
Figure S56.	HMBC spectrum of **6** in C_5D_5N at 298K	37
Figure S57.	IR spectrum of **7**	38
Figure S58.	UV spectrum of **7**	38
Figure S59.	HRESI spectrum of **7**	39
Figure S60.	$[\alpha]^{23}_{D}$ of **7**	39
Figure S61.	^{1}H NMR spectrum of **7** in C_5D_5N at 298K (600 MHz)	40
Figure S62.	^{13}C NMR spectrum of **7** in C_5D_5N at 298K (150 MHz)	40
Figure S63.	HSQC spectrum of **7** in C_5D_5N at 298K	41
Figure S64.	HMBC spectrum of **7** in C_5D_5N at 298K	41
Figure S65.	IR spectrum of **8**	42
Figure S66.	UV spectrum of **8**	42
Figure S67.	HRESI spectrum of **8**	43
Figure S68.	$[\alpha]^{23}_{D}$ of **8**	43
Figure S69.	^{1}H NMR spectrum of **8** in C_5D_5N at 298K (600 MHz)	44
Figure S70.	^{13}C NMR spectrum of **8** in C_5D_5N at 298K (150 MHz)	44
Figure S71.	HSQC spectrum of **8** in C_5D_5N at 298K	45
Figure S72.	HMBC spectrum of **8** in C_5D_5N at 298K	45

Supplementary material

Detailed structural elucidation of 1-8

The molecular formula of **1** was determined as $C_{47}H_{82}O_{18}$ by (+)-HRMS-ESI spectrum at *m/z* 957.5231 [M + Na]$^+$ (calcd. For. $C_{47}H_{82}O_{18}Na$: 957.5542), indicating for the the presence of seven double bonds equivalents (DBEs). The UV bonds at 192. The ^{1}H NMR spectrum of **1** displayed signal for eight methyl protons. The ^{13}C-NMR (Table S1.) revealed forty-seven carbons signals, including four characteristic signals (δ_C: 88.6, 18.2, 70.2, 83.0). With the aid of HMBC spectra, the ^{1}H and ^{13}C NMR spectrum data of **1** was assigned (Table S1).

The presence of HMBC correlations between H_2-23/H_3-26/H_3-27 and C-24, H_3-26/H_3-27 and C-25 indicated a hydroxyl was located at C-25. Besides, in its HMBC spectrum, Glc H-1' (δ_H: 4.97) was correlated with C-3 (δ_C: 88.6). Glc H-1'' (δ_H: 5.12) was correlated with C-20 (δ_C: 83.0), and Xyl H-1''' (δ_H: 4.97) was correlated with C-6'' (δ_C: 69.4), respectively (Figure S1). Glc H-1' was connected with C-3, and Glc C-6'' was connected with Xyl C-1''', finally Glc C-1'' was connected with C-20 indicating from above.

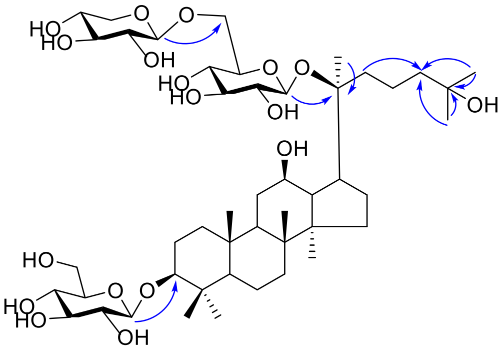

Figure S1. HMBC correlations of **1**

The molecular formula of **2** was deduced as $C_{47}H_{80}O_{18}$ based on a quasi-ion peak at *m/z* 955.5245 [M + Na]$^+$ (calcd. For. $C_{47}H_{80}O_{18}Na$: 955.5232) on its (+)-HRMS-ESI, indicating for the the presence of eight double bonds equivalents (DBEs). The ^{1}H and ^{13}C NMR spectrum data of **2** showed signals for seven methyl protons and forty-seven carbons, including four characteristic signals (δ_C: 88.6, 18.2, 70.1, 83.2) (Table S2).

Besides, analysis of the HMBC spectra, the correlations between H_3-27/H_2-26/H_1-24 and C-25, and δ_H H_2-22/H_2-26/H_3-27 and C-24 showed the existence of a double bond at C-25 and C-26 and a hydroxyl at C-24 showed the existence of a double bond at C-25 and C-26 and a hydroxyl at C-24. In its HMBC spectrum, Glc H-1' (δ_H: 4.97) was correlated with C-3 (δ_C: 88.6). Glc H-1'' (δ_H: 5.17) was correlated with C-20 (δ_C: 83.2), and Ara(f) H-1''' (δ_H: 4.69) was correlated with C-6'' (δ_C: 68.3) (Figure S2), from which revealed Glc H-1' was connected with C-3, and Glc C-6'' was connected with Ara(f) H-1''', finally Glc C-1'' was connected with C-20.

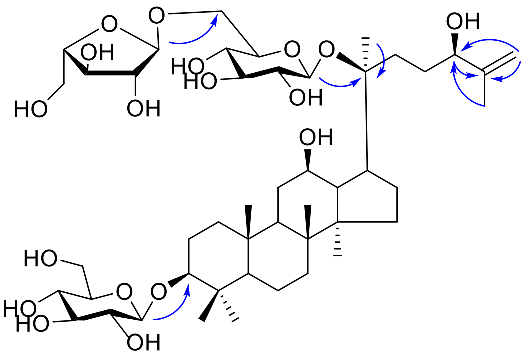

Figure S2. HMBC correlations of **2**

The molecular formula of **3** was determined as $C_{47}H_{80}O_{19}$ based on a quasi-ion peak at *m*/*z* 971.5247 [M + Na]$^{+}$ (calcd. For. $C_{47}H_{80}O_{19}Na$: 971.5242) on its (+)-HRMS-ESI, indicating for the the presence of eight double bonds equivalents (DBEs). The ^{1}H and ^{13}C NMR spectrum data of **3** showed signals for seven methyl protons and forty-seven carbons signals, including three characteristic signals (δ_C: 88.6, 18.2, 83.0) (Table S3).

The HMBC correlations from H_2-26/H_3-27 to C-25, and H_2-23/H_2-26/H_3-27 to C-24 verified that an alkene proton signal existed between C-25 and C-26. Furthermore, according to the analysis of its HMBC spectrum, Glc H-1' (δ_H: 4.97) was correlated with C-3 (δ_C: 88.6). Glc H-1'' (δ_H: 5.12) was correlated with C-20 (δ_C: 83.0), and Xyl H-1''' (δ_H: 4.97) was correlated with C-6'' (δ_C: 69.4) (Figure S3), from which indicated Glc H-1' was connected with C-3, and Glc C-6'' was connected with Xyl H-1''', finally Glc C-1'' was connected with C-20.

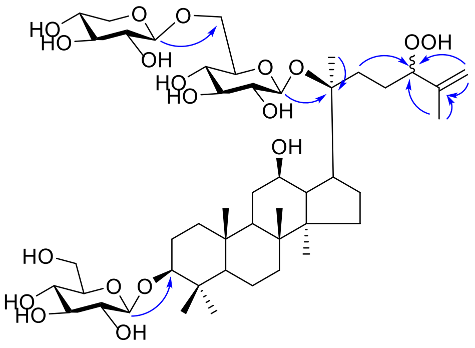

Figure S3. HMBC correlations of **3**

The molecular formula of **4** was determined as $C_{47}H_{80}O_{18}$ based on a quasi-ion peak at *m*/*z* 955.5247 [M + Na]$^{+}$ (calcd. For. $C_{47}H_{80}O_{18}Na$: 955.5641) on its (+)-HRMS-ESI, indicating for the the presence of eight double bonds equivalents (DBEs). The ^{1}H and ^{13}C NMR spectrum data of **4** indicated signals for eight methyl protons and forty-seven carbons signals, including three characteristic signals (δ_C: 89.2, 18.5, 83.8). With the aid of HMBC spectra, the ^{1}H and 13^{C} NMR spectramdata of **4** was assigned (Table S4).

In the HMBC spectrum, H-23/H-24/H_3-26/H_3-27 were correlated with C-25, and H_2-22/H_1-23/H_3-26/H_3-27 were correlated with C-24, indicating that an alkene proton signal existed between C-23 and C-24, and a hydroxyl at C-25. Besides, the HMBC correlations from Glc H-1' (δ_H: 4.93) to C-3 (δ_C: 89.2), Glc H-1'' (δ_H: 5.14) to C-20 (δ_C: 83.8), and Ara(p) H-1''' (δ_H: 5.00) to Glc C-6'' (δ_C: 69.1) (Figure S4) showed Glc H-1' was connected with C-3, and Glc C-6'' was connected with Ara(p) H-1''', finally Glc C-1'' was connected with C-20.

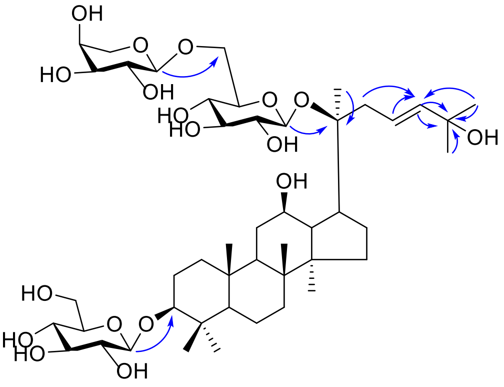

Figure S4. HMBC correlations of **4**

The molecular formula of **5** was deduced as $C_{58}H_{98}O_{28}$ based on a quasi-ion peak at *m/z* 1265.6144 $[M + Na]^+$ (calcd. For. $C_{58}H_{98}O_{28}Na$: 1265.6142) on its (+)-HRMS-ESI, indicating for the the presence of ten double bonds equivalents (DBEs). The ^1H and ^{13}C NMR spectrum data of **5** indicated signals for seven methyl protons and fifty-eight carbons signals, including four characteristic signals (δ_C: 88.6, 18.2, 69.9 and 83.2) (Table S5).

In the HMBC spectrum, Glc H-1' (δ_H: 4.95) was correlated with C-3 (δ_C 88.6), Glc H-1'' (δ_H: 5.54) was correlated with Glc C-2' (δ_C: 82.6), Xyl H-1''' (δ_H: 5.44) was correlated with C-2'' (δ_C: 84.2), Glc H-1'''' (δ_H: 5.12) was correlated with C-20 (δ_C: 83.0), and Xyl H-1''''' (δ_H 5.01) was correlated with Glc C-6'''' (δ_C: 69.9) (shown in Fig 2.), respectively, from which indicated Glc C-1' was connected with C-3, Glc C-1'' was connected with Glc C-2', Glc C-1'''was connected with Xyl C-2'', and Glc C-1''''was connected with C-20, finally Glc C-6'''' was connected with Xyl C-1'''''.

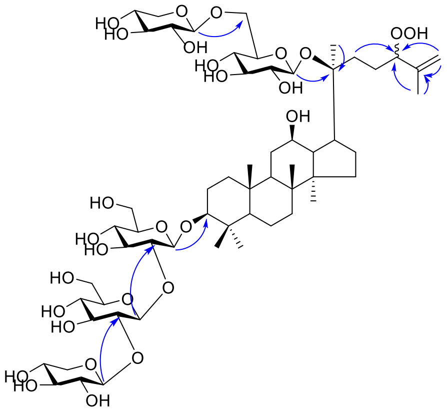

Figure S5. HMBC correlations of **5**

The molecular formula of **6** was determined as $C_{58}H_{98}O_{28}$ based on a quasi-ion peak at *m*/*z* 1265.6149 [M + Na]$^{+}$ (calcd. For. $C_{58}H_{98}O_{28}Na$: 1265.6142) on its (+)-HRMS-ESI, indicating for the the presence of ten double bonds equivalents (DBEs). The ^{1}H and ^{13}C NMR spectrum data of **6** indicated signals for eight methyl protons and fifty-eight carbons signals, including four characteristic signals (δ_C: 88.6, 18.2, 70.2 and 83.0). With the aid of HMBC spectra, the ^{1}H and ^{13}C NMR spectrum data of **6** was assigned (Table S6).

As shown in its HMBC spectrum, H-26/H-27/H-24/H-23 were correlated with C-25, and H-27/H-26/H-23/H-22 were correlated with C-24, indiacting that an alkene proton signal existed between C-23 and C-24, and a a hydroxyperoxy existed at C-25. Besides, the HMBC correlations from Glc H-1' (δ_H: 4.93) to C-3 (δ_C: 89.2). Glc H-1'' (δ_H: 5.14) to C-20 (δ_C: 83.8), and Ara (p) H-1''' (δ_H: 5.00) to Glc C-6'' (δ_C: 69.1) (Figure S6), showed Glc H-1' was connected with C-3, and Glc C-6'' was connected with Ara(p) H-1''', finally Glc C-1'' was connected with C-20.

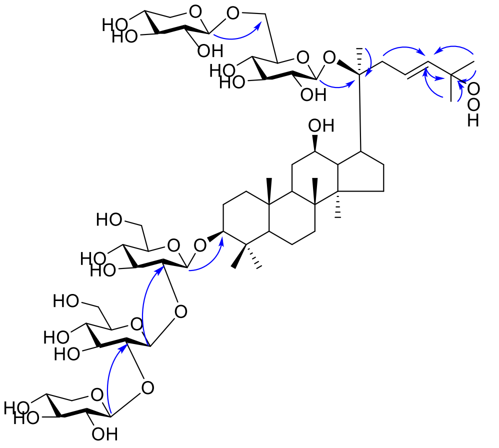

Figure S6. HMBC correlations of **6**

The molecular formula of **7** was deduced as $C_{58}H_{98}O_{28}$ based on a quasi-ion peak at *m*/*z* 1265.6146 [M + Na]$^{+}$ (calcd. For. $C_{58}H_{98}O_{28}Na$: 1265.6146) on its (+)-HRMS-ESI, revealing for the the presence of ten double bonds equivalents (DBEs). The ^{1}H and ^{13}C-NMR spectrum data of **7** indicated signals for eight methyl protons and fifty-eight carbons signals, including four characteristic signals (δ_C: 88.6, 18.2, 70.5 and 83.5) (Table S7).

According to the HMBC spectrum, the correlations from H-26/H-27/H-24/H-23 to C-25, and H-27/H-26/H-23/H-22 to C-24 verified that an alkene proton signal existed between C-23 and C-24, and a a hydroxyperoxy existed at C-25. Besides, Glc H-1' (δ_H: 4.95) was correlated with C-3 (δ_C: 88.6). Glc H-1'' (δ_H: 5.52) was correlated with Glc C-2' (δ_C: 82.7). Xyl H-1''' (δ_H: 5.45) was correlated with Glc C-2'' (δ_H: 84.2). Glc H-1'''' (δ_H: 5.20) was correlated with C-20 (δ_C: 83.3), and Ara (p) H-1''''' (δ_H: 5.00) was correlated with Glc C-6'''' (δ_C 68.7) (Figure S7), indicating Glc H-1' was connected with C-3, Glc C-1'' was correlated with Glc C-2', Glc C-1''' was connected with Xyl C-2'', and Glc C-1'''' was connected with C-20, finally Glc C-6'''' was connected with Ara (p) H-1''''' from above.

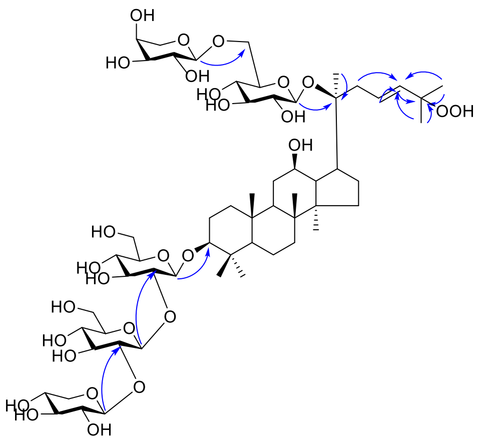

Figure S7. HMBC correlations of **7**

The molecular formula of **8** was determined as $C_{58}H_{96}O_{26}$ based on a quasi-ion peak at *m/z* 1232.4306 [M + Na] $^{+}$ (calcd. For. $C_{58}H_{96}O_{26}Na$: 1232.3532) on its (+)-HRMS-ESI, indicating for the the presence of ten double bonds equivalents (DBEs). The 1H and 13C NMR spectrum data of **8** indicated signals for eight methyl protons and fifty-eight carbons signals, including 28 carbons assigned to the sugar moiety and 30 to a triterpene moiety. With the aid of HMBC spectra, the ^{1}H and ^{13}C NMR spectrum data of **8** was assigned (Table S8).

As shown in its HMBC spectrum, H-22/H-23/H-26/H-27 was correlated with C-24, and H-25/H-26/H-27 was correlated with C-25 were correlated with C-24, indicating that an alkene proton signal existed between C-24 and C-25. Besides, in the HMBC, Glc H-1' (δ_H: 4.96) was correlated with C-3 (δ_C: 89.1). Glc H-1'' (δ_H: 5.40) was correlated with Glc C-2' (δ_C: 83.2), Xyl H-1''' (δ_H: 5.44) was correlated with Glc C-2'' (δ_H: 84.2), and Glc H-1'''' (δ_H: 5.18) was correlated with C-20 (δ_C: 83.3), and Ara (f) H-1''''' (δ_H: 5.68) was correlated with Glc C-6'''' (δ_C: 67.29) (Figure S8), showed Glc C-1' was connected with C-3, Glc C-1'' was connected with Glc C-2', Glc C-1''' was connected with Xyl C-2'', and Glc C-1'''' was connected with C-20, finally Glc C-6'''' was connected with Ara (f) C-1'''''.

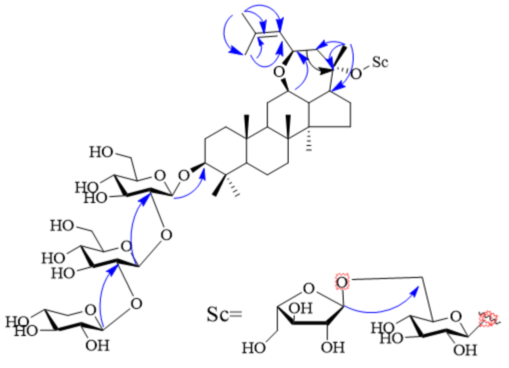

Figure S8. HMBC correlations of **8**

Table S1. ^{1}H and ^{13}C NMR spectral data of **1** (in C_5D_5N, *J* in Hz)

No.	δ_C	δ_H (*J* in Hz)	No.	δ_C	δ_H (*J* in Hz)
1	38.9	0.73 m, 1.54 m	3-O-Glc		
2	26.5	1.80 m, 2.20 m	1'	106.8	4.97 (d, *J* = 7.8 Hz)
3	88.6	3.43 (dd, *J* = 4.8, 10.8 Hz)	2'	75.6	4.08 m
4	39.4	/	3'	78.7	4.19 m
5	56.1	0.70 (d, *J* = 11.4 Hz)	4'	71.6	4.24 m
6	18.2	1.34 m, 1.46 m	5'	78.2	4.04 m
7	34.8	1.46 m[a], 1.18 (d, *J* = 9.6	6'	62.8	4.44 (dd, *J* = 6, 12 Hz)
8	39.7	/	20-O-Glc		
9	49.8	1.36 m	1''	97.9	5.12 (d, *J* = 7.8 Hz)
10	37.3	/	2''	74.8	3.93 m
11	30.7	1.51 m[a], 1.97 m[a]	3''	78.5	4.25 m
12	70.2	3.65 m[a]	4''	71.3	4.20 m
13	49.4	2.00 m	5''	76.6	4.14 m
14	51.2	/	6''	69.4	4.31 m, 4.71 (d, *J* = 11.4 Hz)
15	30.3	0.95 m, 1.83 m	-Xyl		
16	26.1	1.30 m, 1.51 m	1'''	105.5	4.97 (d, *J* = 7.2 Hz)
17	51.6	1.42 m	2'''	74.7	4.02 m[a]
18	15.8	0.90 s	3'''	77.8	4.13 m[a]
19	16.1	0.81 s	4'''	70.9	4.14 m[a]
20	83.0	/	5'''	66.8	3.96 (t, *J* = 10.2 Hz), 4.30 m
21	23.6	1.61 s			
22	39.8	2.80 (dd, *J* = 7.8, 13.8 Hz)			
23	26.4	1.93 m, 2.45 m			
24	27.5	1.81 m, 2.05 m			
25	70.5	/			
26	24.8	1.59 s			
27	25.2	1.53 s			
28	25.9	1.30 s			
29	16.6	0.99 s			
30	17.0	1.00 s			

a: Overlapped signals, s: singlet, d: doublet, t: triplet, m: multiplet, ′: the first sugar, ″: the second sugar, /: no hydrogen.

Table S2. ^{1}H and ^{13}C NMR spectral data of **2** (in C_5D_5N, *J* in Hz)

No.	δ_C	δ_H (*J* in Hz)	No.	δ_C	δ_H (*J* in Hz)
1	38.9	0.76 m, 1.52 m	3-O-		
2	26.5	1.82 m, 2.28 m	1'	106.8	4.97 (d, *J* = 7.8 Hz)
3	88.6	3.37 (dd, *J* = 4.8, 10.8 Hz)	2'	76.0	4.08 m
4	39.4	/	3'	78.9	4.21 m
5	56.1	0.72 m	4'	71.8	3.99 m
6	18.2	1.36 m, 1.47 m	5'	78.2	4.05 m
7	34.8	1.20 m, 1.48m	6'	62.8	4.44 (dd, *J* = 4.8, 10.8 Hz)
8	39.7	/	20-O-		
9	49.9	1.37 m	1''	97.9	5.17 (d, *J* = 7.7 Hz)
10	36.7	/	2''	75.1	3.97 m
11	30.1	1.40 m, 1.99 m[a]	3''	78.8	4.30 m
12	70.1	3.12 m	4''	71.9	4.22 m
13	49.2	2.05 m	5''	76.4	4.04 m
14	51.2	/	6''	68.3	4.09 m, 4.36 m
15	30.5	0.97 m, 1.45 m	-		
16	26.4	1.38 m, 2.24 m	1'''	109.8	4.69 br.s
17	51.7	2.54 m	2'''	83.4	4.13 m[a]
18	15.6	0.93 s	3'''	78.7	4.16 m[a]
19	16.0	0.79 s	4'''	85.9	4.06 m[a]
20	83.2	/	5'''	62.7	3.71 (d, *J* = 10.2 Hz),
21	22.3	1.63 s			4.30 m
22	32.6	2.54 m, 2.24 (dd, J = 9.8, 15.2 Hz)			
23	30.6	1.53 m, 2.23 m			
24	76.6	4.47 m[a]			
25	150.0	/			
26	110.3	4.95 br.s, 5.26 br.s			
27	17.8	1.971 s			
28	27.9	1.31 s			
29	16.6	0.96 s			
30	17.0	0.94 s			

a: Overlapped signals, s: singlet, d: doublet, m: multiplet, br.s: broad singlet, ′: the first sugar, ″: the second sugar, ‴: the third sugar, /: no hydrogen.

Table S3. ^{1}H and ^{13}C NMR spectral data of **3** (in C_5D_5N, *J* in Hz)

No.	δ_C	δ_H (*J* in Hz)	No.	δ_C	δ_H (*J* in Hz)
1	38.9	0.76 m, 1.52 m	3-O-Glc		
2	26.5	1.82 m, 2.28 m	1'	106.8	4.97 (d, *J* = 7.6 Hz)
3	88.6	3.38 (dd, *J* = 4.2, 11.6 Hz)	2'	75.6	4.08 m
4	39.4	/	3'	78.7	4.19 m
5	56.1	0.73 (d, *J* = 12.4 Hz)	4'	71.6	4.24 m
6	18.2	1.36 m, 1.47 m	5'	78.2	4.04 m
7	34.8	1.18 m	6'	62.8	4.64 (d, *J* =12.4 Hz)
8	39.7	/	20-O-Glc		
9	50.0	1.37 m	1''	97.6	5.12 br.s
10	37.3	/	2''	74.8	3.93 m
11	30.7	1.51 m[a], 1.99 m[a]	3''	78.5	4.25 m
12	66.9	4.18 m[a]	4''	71.3	4.20 m[a]
13	49.3	2.02 m	5''	76.6	4.14 m[a]
14	51.2	/	6''	69.4	4.10 m, 4.31 m
15	30.3	0.95 m[a], 1.45 m[a]	-Xyl		
16	26.1	1.30 m[a], 2.25 m[a]	1'''	105.5	4.97 (d, *J* = 7.2 Hz)
17	51.6	1.42 m[a]	2'''	74.7	4.02 m
18	15.8	0.97 s	3'''	77.8	4.13 m
19	16.1	0.81 s	4'''	70.9	4.14 m
20	83.0	/	5'''	66.8	3.96 (t, *J* = 10.2 Hz), 4.30 m
21	23.6	1.63 s			
22	22.2	1.64 m[a]			
23	26.0	1.97 m[a], 2.23 m[a]			
24	89.8	4.80 m			
25	145.9	/			
26	110.3	5.09 br.s, 5.27 br.s			
27	17.3	1.96 s			
28	27.9	1.32 s			
29	16.6	1.01 s			
30	17.0	0.98 s			

a: Overlapped signals, s: singlet, d: doublet, t: triplet, m: multiplet, br.s: broad singlet, ′: the first sugar, ″: the second sugar, ‴: the third sugar, /: no hydrogen.

Table S4. ^1H and ^{13}C NMR spectral data of **4** (in C_5D_5N, *J* in Hz)

No.	δ_C	δ_H (*J* in Hz)	No.	δ_C	δ_H (*J* in Hz)
1	39.1	0.73 m, 1.52 m	3-O-Glc		
2	26.8	1.34 m, 1.98 m	1'	104.7	4.93 (d, *J* = 7.8 Hz)
3	89.2	3.43 (dd, *J* =4.8, 10.8 Hz)	2'	76.0	4.08 m[a]
4	39.7	/	3'	78.9	4.21 m[a]
5	56.4	0.66 m	4'	71.8	3.99 m
6	18.5	1.34 m, 1.47 m	5'	78.2	4.05 m
7	35.1	1.19 m[a]	6'	62.8	4.44 m
8	40.1	/	20-O-Glc		
9	50.1	1.35 m	1''	97.9	5.14 (d, *J* = 7.8 Hz)
10	36.9	/	2''	74.8	3.93 m
11	30.8	1.54 m[a], 1.99 m[a]	3''	78.6	4.35 m
12	70.6	3.65 m	4''	71.0	3.96 m[a]
13	49.5	4.94 m	5''	76.7	4.04 m
14	51.5	/	6''	69.1	4.15 m, 4.25 m
15	30.5	0.97 m[a], 1.45 m[a]	-Ara(p)		
16	26.4	1.38 m[a], 2.25 m[a]	1'''	104.6	5.00 (d, *J* = 6.0 Hz)
17	52.4	3.16 m	2'''	72.1	4.46 m
18	16.6	0.87 s	3'''	74.1	4.22 m
19	16.3	0.95 s	4'''	68.5	4.37 m
20	83.8	/	5'''	65.6	3.79 m, 4.30 m
21	23.3	1.48 s			
22	39.6	2.24 (dd, *J* =16.0, 9.6 Hz)			
23	122.7	6.23 (ddd, *J* = 5.8, 8.5,15.5 Hz)			
24	142.2	6.09 (d, *J* = 15.5 Hz)			
25	70.0	/			
26	17.7	1.59 s			
27	17.8	1.53 s			
28	27.9	1.30 s			
29	28.1	0.99 s			
30	17.2	1.02 s			

a: Overlapped signals, s: singlet, d: doublet, m: multiplet, br.s: broad singlet, ': the first sugar, '': the second sugar, ''': the third sugar, /: no hydrogen.

Table S5. ^{1}H and ^{13}C NMR spectral data of **5** (in C_5D_5N, J in Hz)

No.	δ_C	δ_H (J in Hz)	No.	δ_C	δ_H (J in Hz)
1	39.0	0.74 m, 1.52 m	3-O-Glc		
2	26.6	1.34 m, 1.82 m	1'	104.6	4.95 (d, J = 7.7 Hz)
3	88.6	3.30 (dd, J = 3.9, 11.5 Hz)	2'	82.3	4.13 m[a]
4	39.5	/	3'	78.5	4.37 m[a]
5	56.1	0.68 d (J = 11.9 Hz)	4'	71.4	4.11 m[a]
6	18.2	1.34 m, 1.47 m	5'	78.1	3.99 m[a]
7	34.9	1.20 d (J = 11.9 Hz)	6'	62.7	4.39 m[a]
8	39.8	/	-O-Glc		
9	50.0	1.35 m[a]	1''	103.0	5.54 (d, J = 6.8 Hz)
10	36.7	/	2''	84.2	4.23 m[a]
11	30.7	1.51 m[a], 1.89 m[a]	3''	77.7	4.31 m[a]
12	69.9	4.18 m[a]	4''	71.1	4.24 m[a]
13	49.3	2.00 m[a]	5''	77.6	4.16 m[a]
14	51.1	/	6''	62.5	4.39 m[a]
15	30.4	0.96 m[a], 1.42 m[a]	-Xyl		
16	26.4	1.34 m[a], 2.30 m[a]	1'''	106.2	5.44 (d, J = 6.8 Hz)
17	51.2	2.57 m[a]	2'''	75.8	4.13 m[a]
18	15.8	0.96 s	3'''	78.5	4.37 m[a]
19	16.1	0.80 s	4'''	70.9	4.11 m[a]
20	83.2	/	5'''	67.2	3.71 (t, J = 10.2 Hz)
21	22.1	1.64 s	20-O-Glc		
22	32.7	1.90 m, 2.58 m	1''''	97.8	5.12 br.s
23	26.4	1.97 m[a], 2.20 m[a]	2''''	74.7	3.88 m[a]
24	90.0	4.80 (t, J = 6.7 Hz)	3''''	78.1	4.17 m[a]
25	146.1	/	4''''	71.5	4.24 m[a]
26	113.1	5.09 br.s, 5.28 br.s	5''''	76.6	3.99 m[a]
27	17.3	1.96 s	6''''	69.9	4.30 m[a]
28	27.8	1.28 s	-Xyl		4.76 (d, J = 10.0 Hz)
29	16.4	1.11 s	1'''''	105.5	5.01 (d, J = 7.4 Hz)
30	17.2	0.95 s	2'''''	74.6	4.06 m[a]
			3'''''	78.4	4.37 m[a]
			4'''''	70.5	4.17 m[a]
			5'''''	66.8	3.76 (t, J = 10.2 Hz)
					4.33 m[a]

a: Overlapped signals, s: singlet, d: doublet, t: triplet, m: multiplet, br.s: broad singlet, ′: the first sugar, ″: the second sugar, ‴: the third sugar, ⁗: the fourth sugar, ″‴: the fifth sugar, /: no hydrogen.

Table S6. 1H and 13C NMR spectral data of **6** (in C5D5N, *J* in Hz)

No.	δC	δH (*J* in Hz)	No.	δC	δH (*J* in Hz)
1	38.9	0.74 m, 1.52 m	3-O-Glc		
2	26.5	1.33 m, 1.81 m	1'	104.6	4.96 (d, *J* = 6.2 Hz)
3	88.6	3.30 (dd-like)	2'	82.7	4.15 m[a]
4	39.5	/	3'	77.7	4.39 m[a]
5	56.1	0.69 (d, *J* = 11.7 Hz)	4'	71.4	4.14 m[a]
6	18.2	1.38 m, 1.54 m[a]	5'	77.9	3.99 m[a]
7	34.8	1.21 (d, *J* = 11.0 Hz)	6'	62.7	4.51 m[a]
8	39.8	/	-O-Glc		
9	49.9	1.38 (d, *J* = 11.7 Hz)	1''	102.9	5.54 (dd-like)
10	36.7	/	2''	84.3	4.24 m[a]
11	30.7	1.54 m[a], 1.90 m[a]	3''	78.1	4.31 m[a]
12	70.2	4.07 m[a]	4''	70.9	4.24 m[a]
13	49.4	2.02 m[a]	5''	77.6	4.16 m[a]
14	51.2	/	6''	62.5	4.03 m, 4.39 m[a]
15	30.3	0.98 m[a], 1.44 m[a]	-Xyl		
16	26.1	1.46 m[a], 2.30 m[a]	1'''	106.2	5.45 (d, *J* = 6.0 Hz)
17	51.6	2.47 m[a]	2'''	75.8	4.14 m[a]
18	15.8	1.01 s	3'''	78.5	4.39 m[a]
19	16.1	0.83 s	4'''	71.3	4.14 m[a]
20	83.0	/	5'''	67.2	3.72 m[a]
21	23.0	1.63 s	20-O-Glc		
22	39.8	2.85 m, 3.13 m[a]	1''''	98.0	5.20 br.s
23	126.5	6.20 m[a]	3''''	78.1	3.95 m[a]
24	137.8	6.15 br.s	4''''	71.5	4.21 m[a]
25	81.1	/	5''''	76.6	3.99 m[a]
26	24.8	1.62 s	6''''	69.7	4.35 m[a]
27	25.2	1.62 s	-Xyl		4.76 (d, *J* = 11.2
28	27.8	1.29 s	1'''''	105.4	5.00 (d, *J* = 6.8 Hz)
29	16.3	1.12 s	2'''''	74.7	4.05 m[a]
30	17.0	0.92 s	3'''''	78.8	4.39 m[a]
			4'''''	70.5	4.17 m[a]
			5'''''	66.8	3.72 m[a], 4.34 m[a]

a: Overlapped signals, s: singlet, d: doublet, m: multiplet, br.s: broad singlet, ': the first sugar, '': the second sugar, ''': the third sugar, '''': the fourth sugar, ''''': the fifth sugar, /: no hydrogen.

Table S7. ^{1}H and ^{13}C NMR spectral data of **7** (in C_5D_5N, *J* in Hz)

No.	δ_C	δ_H (*J* in Hz)	No.	δ_C	δ_H (*J* in Hz)
1	38.9	0.73 m, 1.52 m	3-O-Glc		
2	26.6	1.40 m, 1.95 m	1'	104.6	4.95(d, *J* = 6.2 Hz)
3	88.6	3.30 (dd, *J* = 4.2, 11.4 Hz)	2'	82.7	4.13 m[a]
4	39.5	/	3'	77.6	4.20 m[a]
5	56.1	0.68 (d, *J* = 11.3 Hz)	4'	71.43	4.14 m[a]
6	18.2	1.37 m, 1.55 m[a]	5'	77.6	3.90 m[a]
7	34.9	1.20 (d, *J* = 11.0 Hz)	6'	62.7	4.39 m[a], 4.60 m[a]
8	39.8	/	-O-Glc		
9	49.9	1.38 (d, *J* = 11.7 Hz)	1''	102.9	5.52 (d, *J* = 7.8 Hz)
10	36.7	/	2''	84.2	4.24 m[a]
11	30.7	1.53 m[a], 1.92 m[a]	3''	78.1	3.95 m[a]
12	70.5	4.13 m[a]	4''	70.9	4.07 m[a]
13	49.4	2.00 m[a]	5''	77.6	4.26 m[a]
14	51.2	/	6''	62.5	4.00 m, 4.37 m[a]
15	30.6	0.97 m[a], 1.42 m[a]	-Xyl		
16	26.4	1.80 m[a], 2.47 m[a]	1'''	106.2	5.43 (d, *J* = 6.6 Hz)
17	51.6	2.57 m[a]	2'''	75.8	4.12 m[a]
18	15.8	0.79 s	3'''	78.5	4.39 m[a]
19	16.1	0.93 s	4'''	71.3	4.12 m[a]
20	83.3	/	5'''	67.2	3.70 m[a]
21	22.4	1.63 s	20-O-Glc		
22	39.6	1.85 m	1''''	98.0	5.20 s
23	126.2	5.20 m[a]	2''''	74.8	3.95 m[a]
24	137.8	6.15 br.s	3''''	78.5	4.33 m[a]
25	81.1	/	4''''	71.3	3.92 m[a]
26	24.8	1.61 s	5''''	76.4	3.99 m[a]
27	25.2	1.61 s	6''''	68.7	4.25 m[a]
28	27.8	1.27 s			4.71 (d, *J* = 11.2 Hz)
			-Ara (p)		
29	16.3	1.14 s	1'''''	104.1	5.00 (d, *J* = 6.8 Hz)
30	16.8	0.94 s	2'''''	71.8	4.55 m[a]
			3'''''	73.9	4.23 m[a]
			4'''''	68.3	4.37 m[a]
			5'''''	65.1	3.79 m[a], 4.31 m[a]

a: Overlapped signals, s: singlet, d: doublet, m: multiplet, br.s: broad singlet, ': the first sugar, '': the second sugar, ''': the third sugar, '''': the fourth sugar, ''''': the fifth sugar, /: no hydrogen.

Table S8. ^{1}H and ^{13}C NMR spectral data of **8** (in C_5D_5N, *J* in Hz)

No.	δ_C	δ_H (*J* in Hz)	No.	δ_C	δ_H (*J* in Hz)
1	39.8	0.86 m, 1.56 m	3-O-Glc		
2	26.7	1.76 m, 2.19 m	1'	105.3	4.96 (d, *J* = 8.3 Hz)
3	89.1	3.30 (dd, *J*= 4.2, 11.4 Hz)	2'	83.2	4.23 m[a]
4	39.1	/	3'	77.4	4.30 m[a]
5	56.5	0.61 (d, *J*= 11.9 Hz)	4'	71.4	4.24 m[a]
6	18.5	1.50, 1.40	5'	78.1	3.94 m[a]
7	35.2	1.47, 1.15	6'	62.8	4.47 m[a], 4.34 m[a]
9	50.2	1.51 (d, *J* = 11.7 Hz)	1''	105.6	5.40 (d, *J*= 6.9 Hz)
10	37.0	/	2''	75.2	4.08 m[a]
11	30.9	1.80 m, 1.34 m	3''	77.3	4.37 m[a]
12	79.1	3.72 m[a]	4''	71.7	4.13 m[a]
13	49.5	1.38	5''	78.3	3.99 m[a]
14	51.6	/	6''	62.6	4.47 m, 4.22 m[a]
15	31.1	1.43 m[a], 1.15 m[a]	-Xyl		
16	26.9	2.24 m[a], 2.16 m[a]	1'''	106.2	5.44 (d, *J* = 6.6 Hz)
17	49.8	3.61 m[a]	2'''	75.1	4.30 m[a]
18	15.9	0.88 s	3'''	78.5	4.37 m[a]
19	16.4	0.92 s	4'''	70.4	4. 04 m[a]
20	83.6	/	5'''	65.8	3.75 m[a]
21	22.8	1.61 s	20-O-Glc		
22	52.7	2.16 m, 2.24 m	1''''	98.4	5. 18 br.s
23	71.9	4.83 (t, *J* = 8.2 Hz)	3''''	78.5	3.95 m[a]
24	129.4	6.02 (d, *J* = 7.6 Hz)	4''''	78.3	4.33 m[a]
25	131.6	/	5''''	71.7	4.70 m[a]
26	29.7	1.65 s	6''''	67.2	4.36 m[a], 4.29 m[a]
			-Ara(f)		
27	19.5	1.94 s	1'''''	110.3	5.68 (d, *J* = 6.8 Hz)
28	28.2	1.27 s	2'''''	83.5	4.27 m[a]
29	16.7	1.10 s	3'''''	78.8	4.41 m[a]
30	17.4	0.79 s	4'''''	85.9	4.23 m[a]
			5'''''	62.9	4.40 m[a], 4.30 m[a]

a: Overlapped signals, s: singlet, d: doublet, t: triplet, m: multiplet, br.s: broad singlet, ′: the first sugar, ″: the second sugar, ‴: the third sugar, ⁗: the fourth sugar, ′′′′′: the fifth sugar, /: no hydrogen.

CD, UV, IR, HRESI, and NMR of 1-8

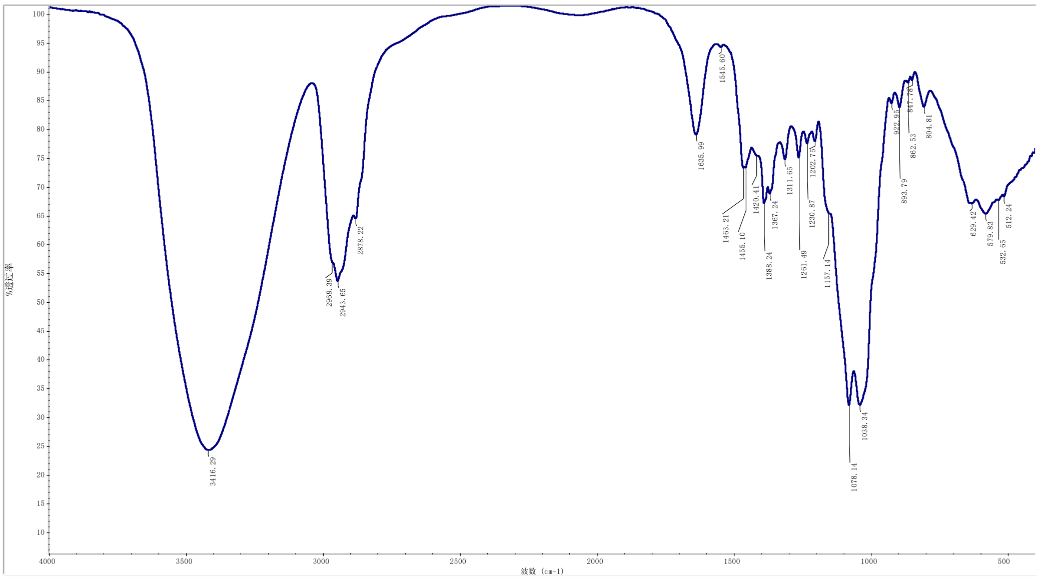

Figure S9. IR spectrum of **1**

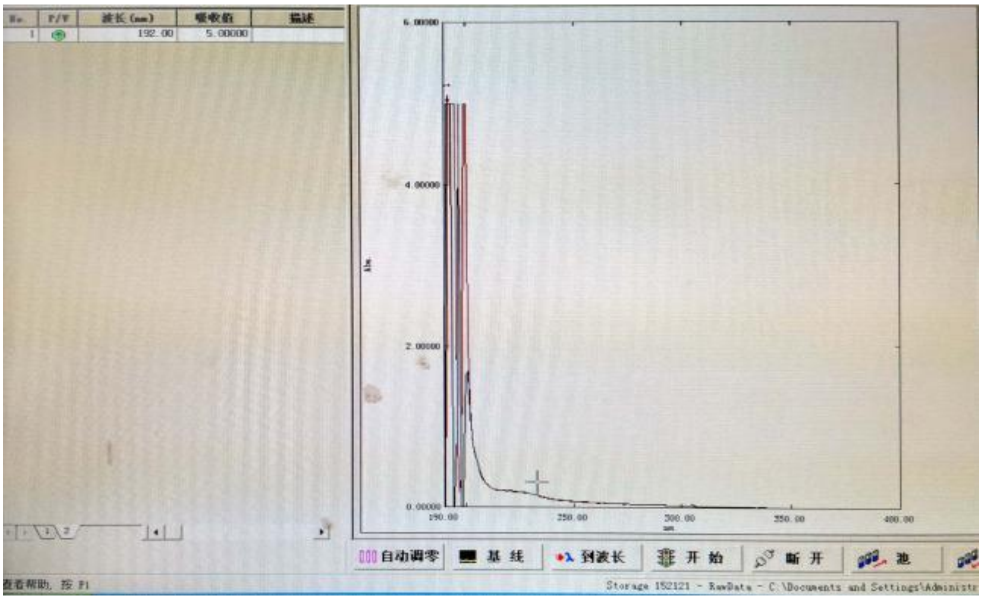

Figure S10. UV spectrum of **1**

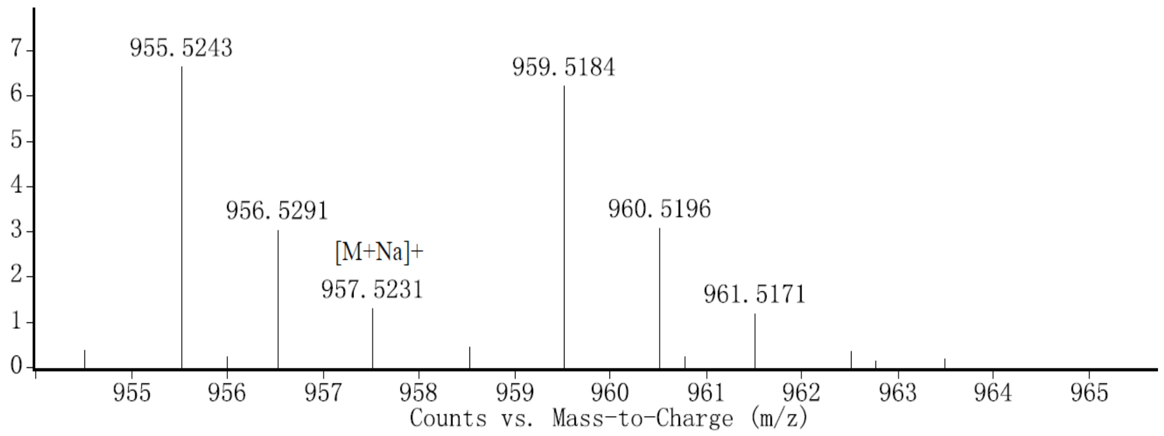

Figure S11. HRESI spectrum of **1**

Rudolph Research Analytical

This sample was measured on an Autopol VI, Serial #91058
Manufactured by Rudolph Research Analytical, Hackettstown, NJ, USA.

Measurement Date : Thursday, 27-FEB-2020

Set Temperature : OFF

Time Delay : Disabled

Delay between Measurement : Disabled

n	Average	Std.Dev.	% RSD	Maximum	Minimum
5	7.00	0.45	6.42	7.50	6.67

S.No	Sample ID	Time	Result	Scale	OR °Arc	WLG.nm	Lg.mm	Conc.g/100ml	Temp.
1	GY-20	02:47:49 PM	7.50	SR	0.009	589	100.00	0.120	23.4
2	GY-20	02:47:55 PM	7.50	SR	0.009	589	100.00	0.120	23.4
3	GY-20	02:48:01 PM	6.67	SR	0.008	589	100.00	0.120	23.3
4	GY-20	02:48:07 PM	6.67	SR	0.008	589	100.00	0.120	23.3
5	GY-20	02:48:12 PM	6.67	SR	0.008	589	100.00	0.120	23.3

Figure S12. $[\alpha]_{D}^{23}$ of **1**

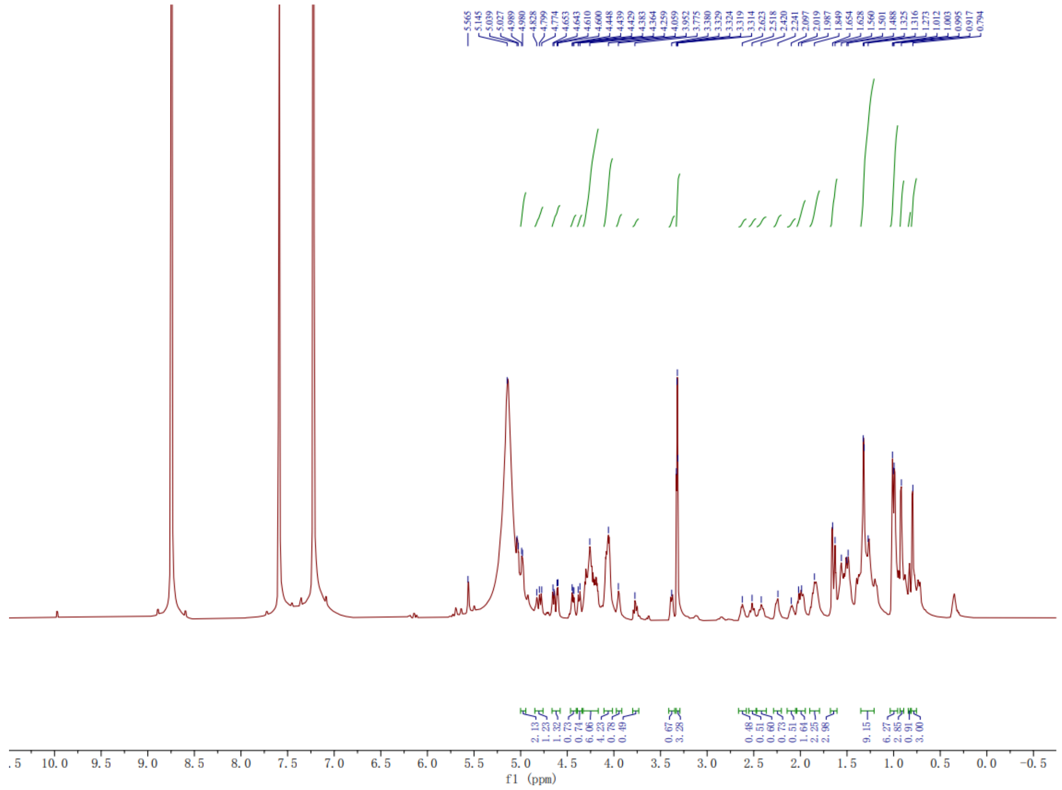

Figure S13. ^{1}H-NMR spectrum of **1** in C_5D_5N at 298K (600 MHz)

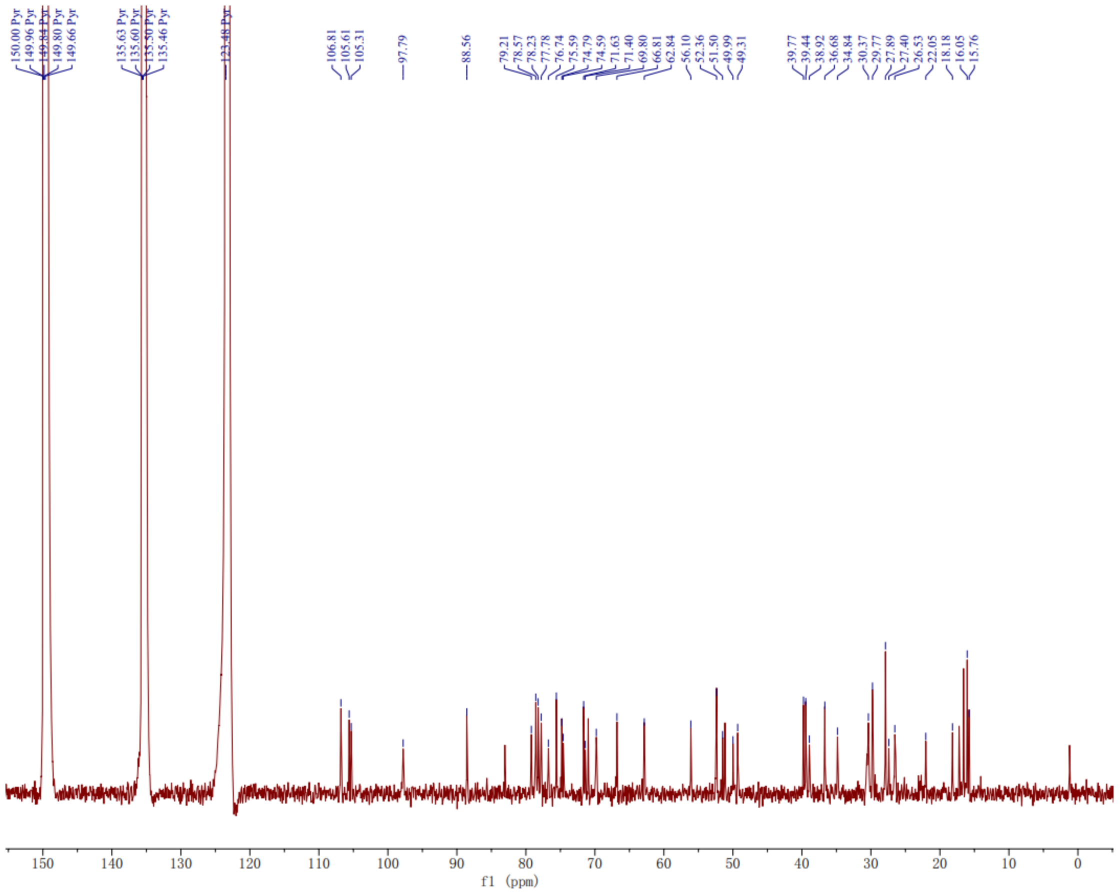

Figure S14. ^{13}C-NMR spectrum of **1** in C_5D_5N at 298K (150 MHz)

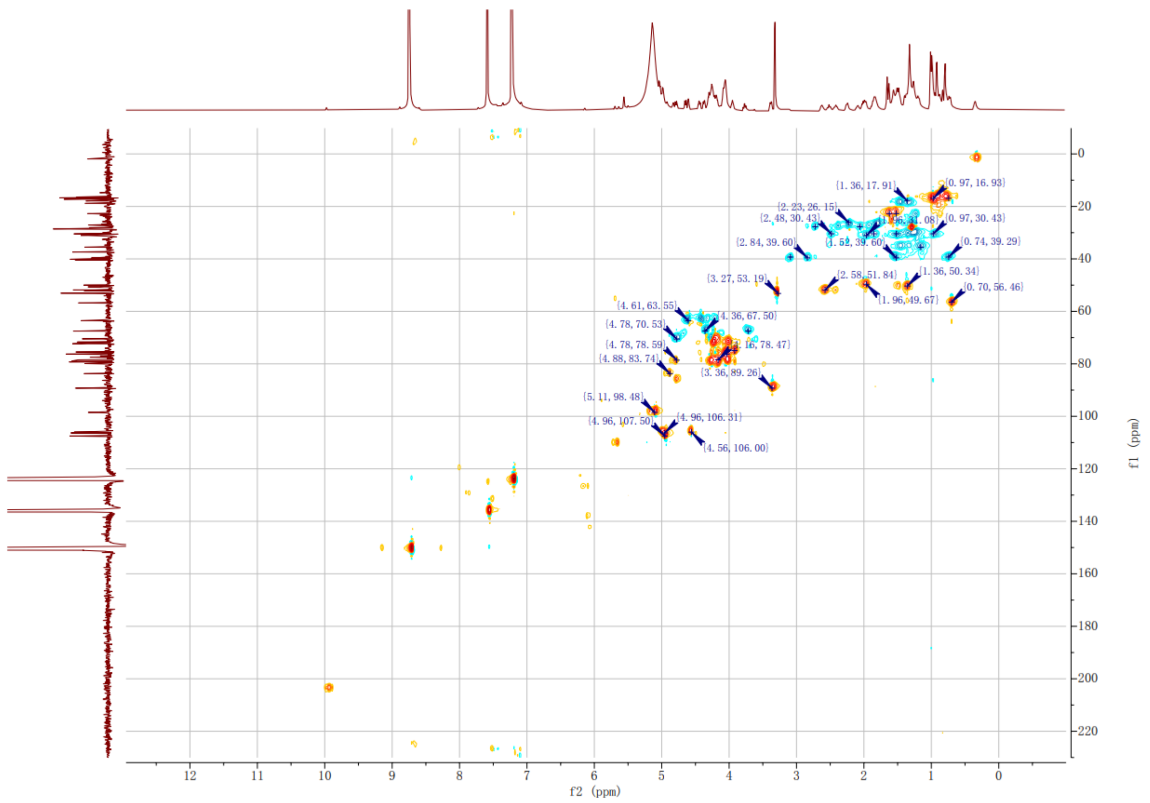

Figure S15. HSQC spectrum of **1** in C_5D_5N at 298K

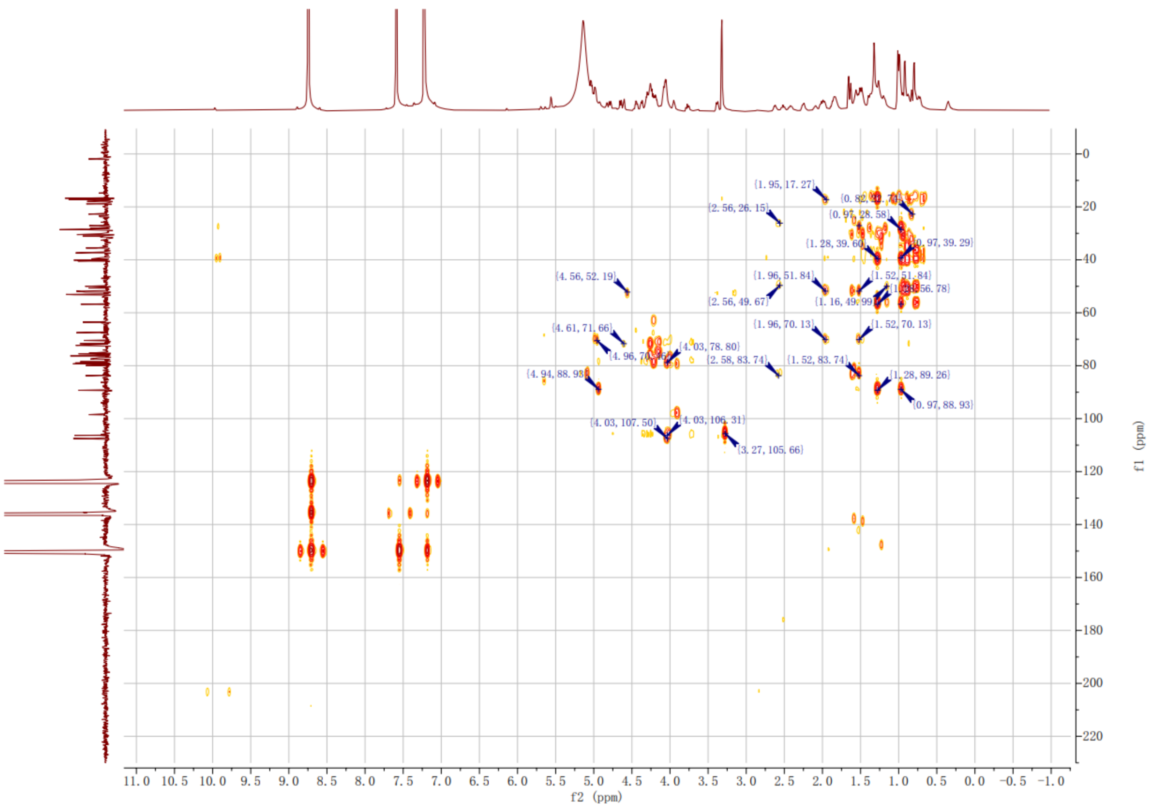

Figure S16. HMBC spectrum of **1** in C_5D_5N at 298K

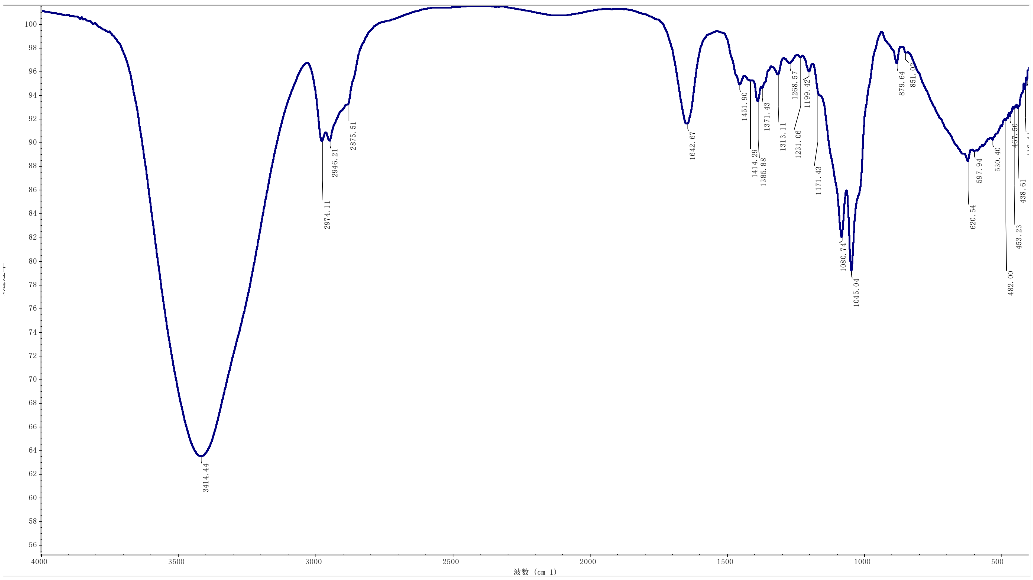

Figure S17. IR spectrum of **2**

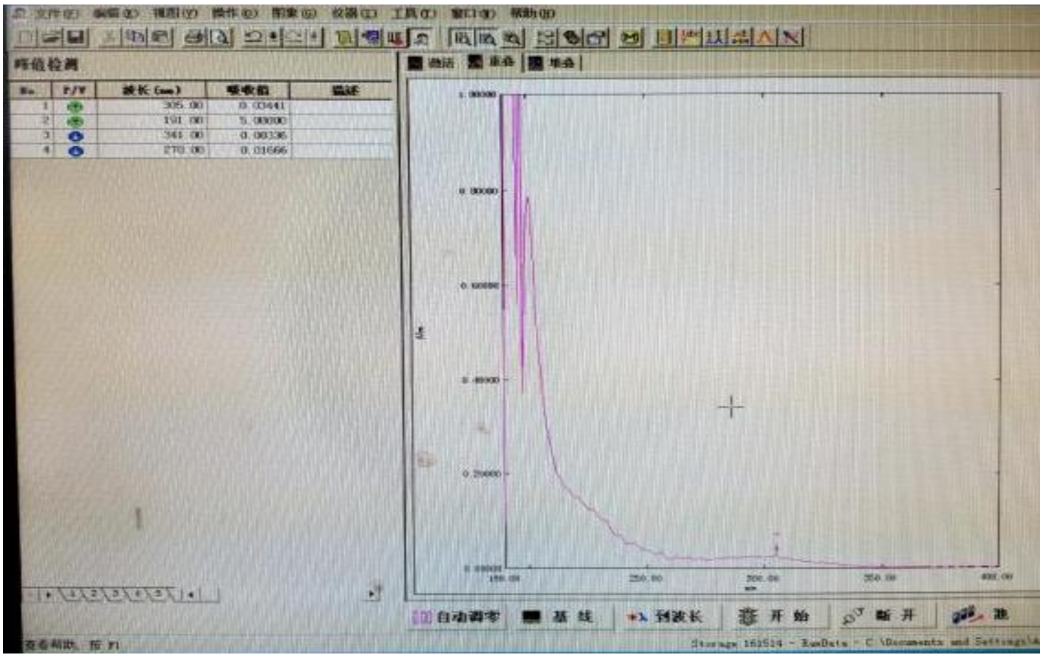

Figure S18. UV spectrum of **2**

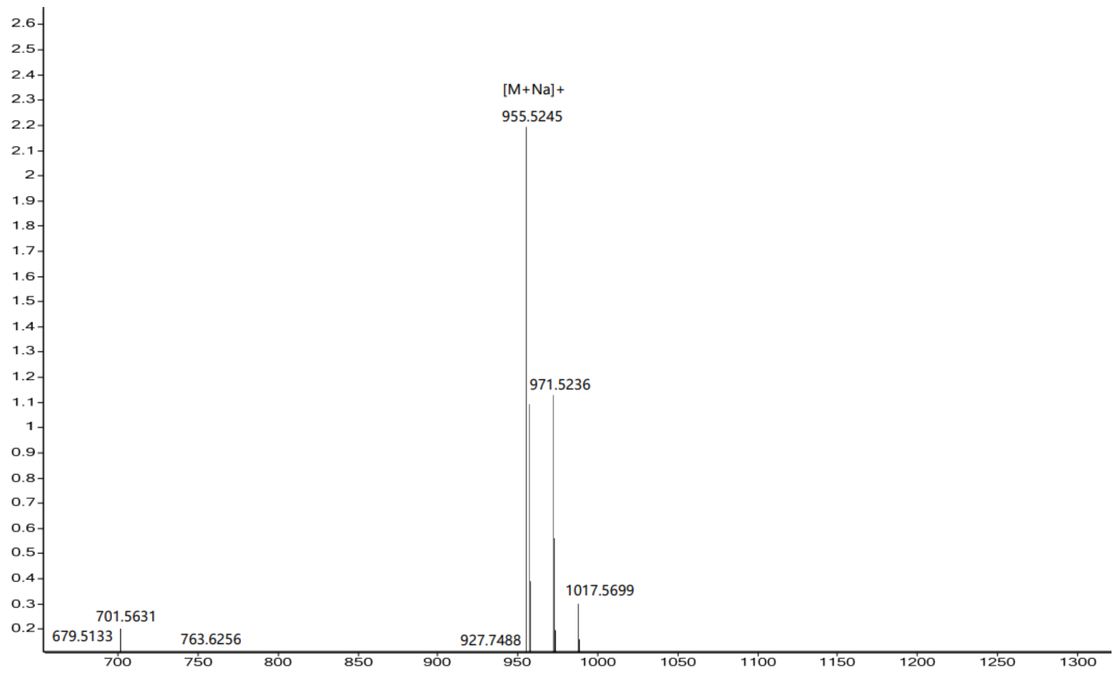

Figure S19. HRESIMS of **2**

<u>**Rudolph Research Analytical**</u>

This sample was measured on an Autopol VI, Serial #91058
Manufactured by Rudolph Research Analytical, Hackettstown, NJ, USA.

Measurement Date : Thursday, 27-FEB-2020

Set Temperature : OFF

Time Delay : Disabled

Delay between Measurement : Disabled

n	Average	Std.Dev.	% RSD	Maximum	Minimum
5	-1.46	0.81	-55.47	-0.91	-2.73

S.No	Sample ID	Time	Result	Scale	OR °Arc	WLG.nm	Lg.mm	Conc.g/100ml	Temp.
1	GY-23	02:51:41 PM	-1.82	SR	-0.002	589	100.00	0.110	23.2
2	GY-23	02:51:46 PM	-0.91	SR	-0.001	589	100.00	0.110	23.2
3	GY-23	02:51:52 PM	-0.91	SR	-0.001	589	100.00	0.110	23.2
4	GY-23	02:51:58 PM	-0.91	SR	-0.001	589	100.00	0.110	23.2
5	GY-23	02:52:03 PM	-2.73	SR	-0.003	589	100.00	0.110	23.2

Figure S20. $[\alpha]_{D}^{23}$ of **2**

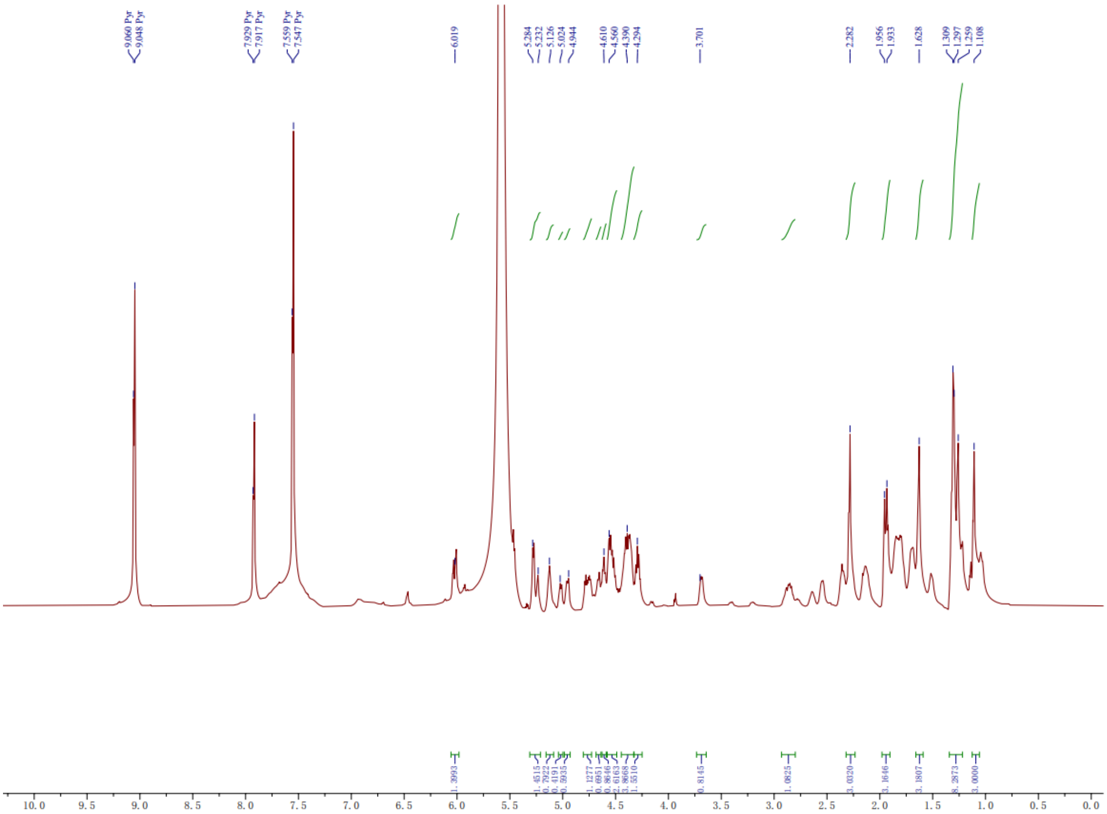

Figure S21. ^{1}H-NMR spectrum of **2** in C_5D_5N at 298K (600 MHz)

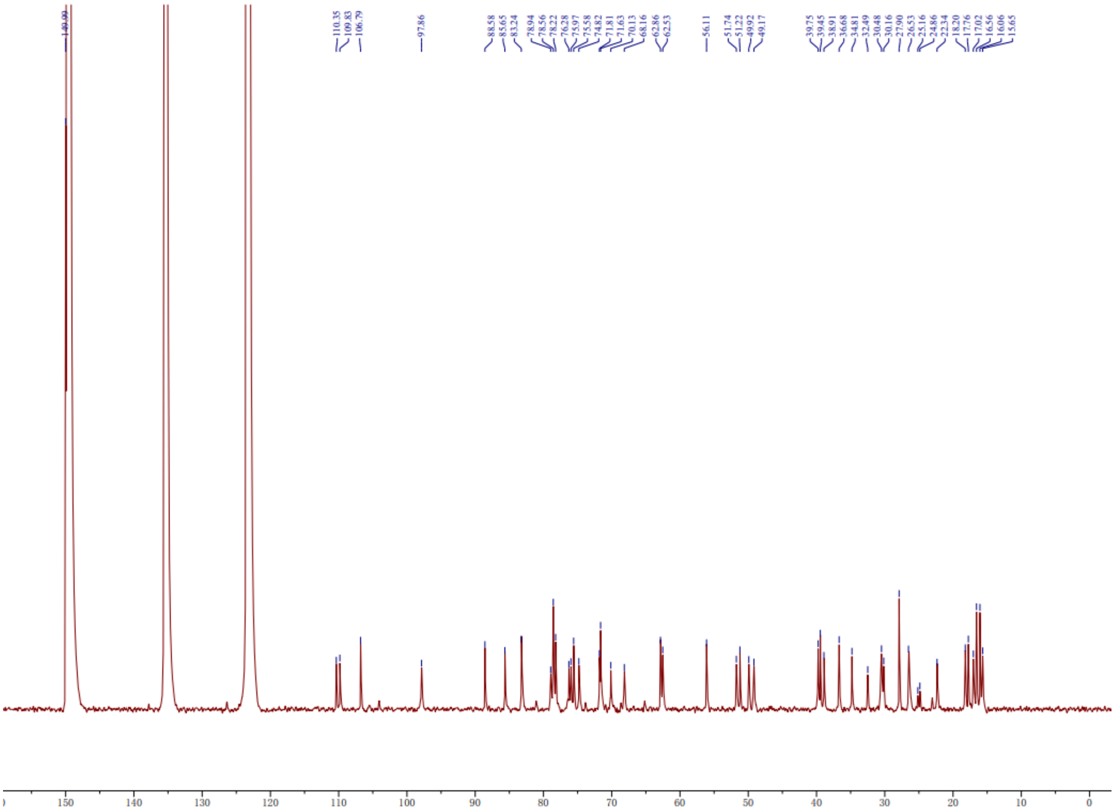

Figure S22. ^{13}C-NMR spectrum of **2** in C_5D_5N at 298K (150 MHz)

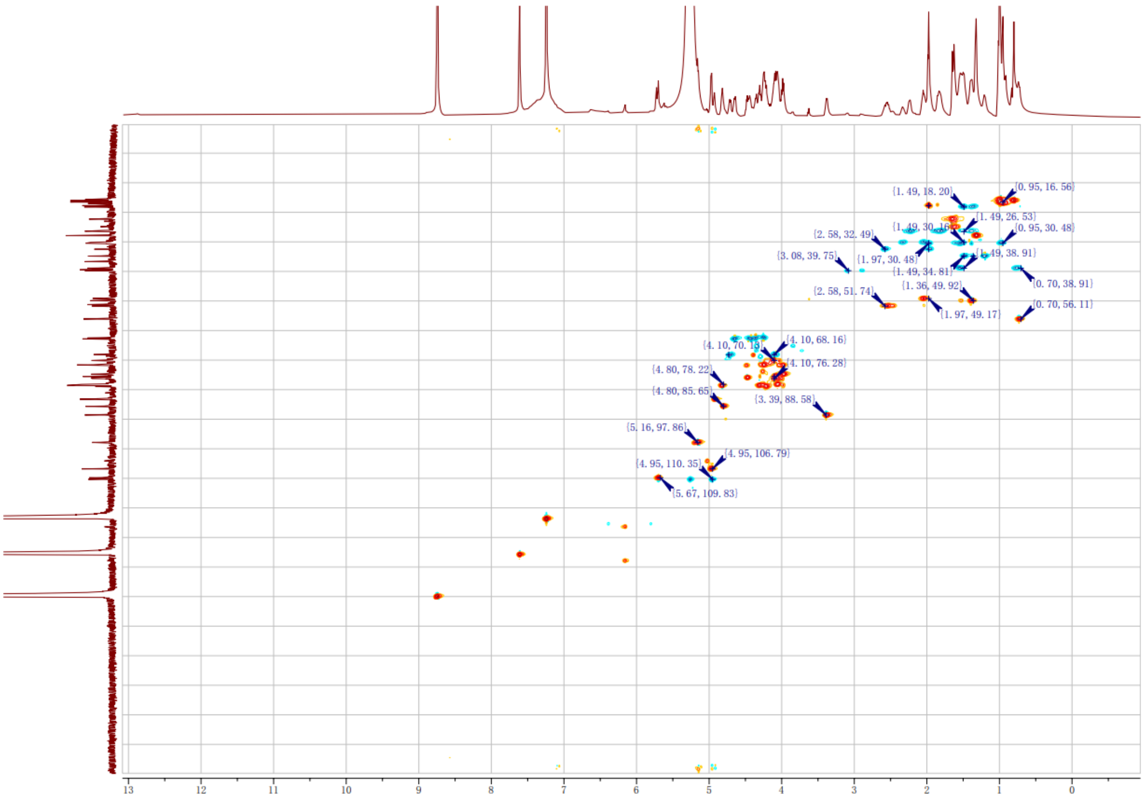

Figure S23. HSQC spectrum of **2** in C_5D_5N at 298K

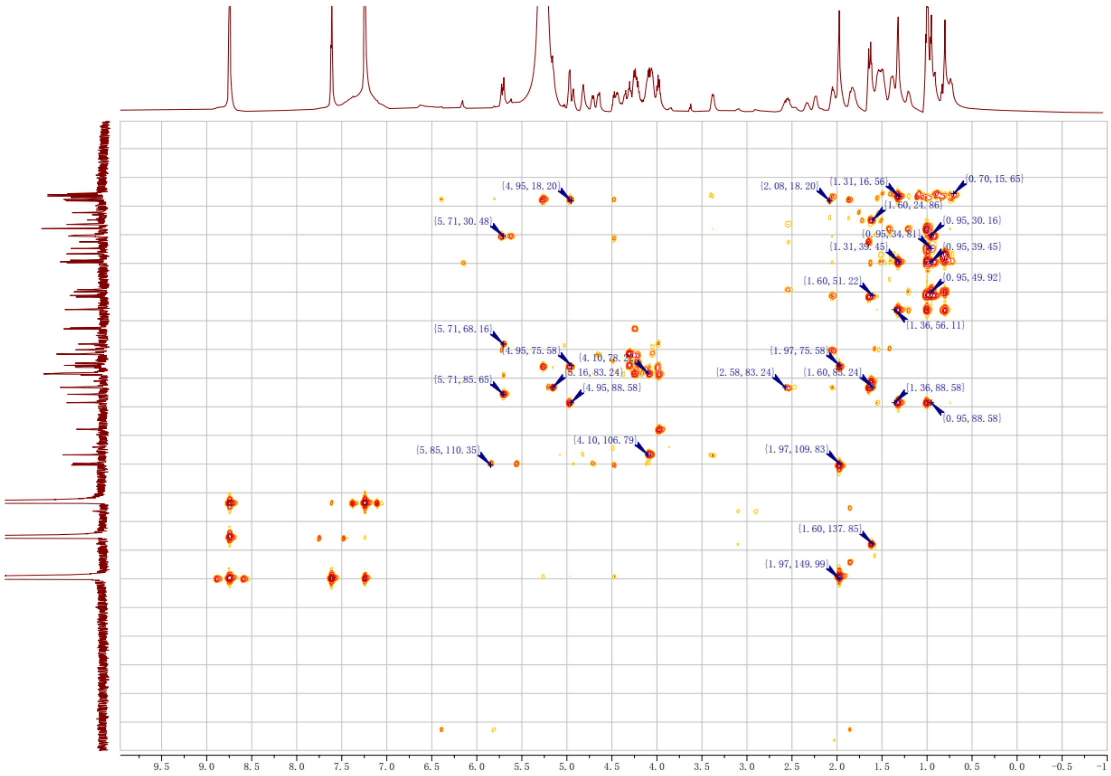

Figure S24. HMBC spectrum of **2** in C_5D_5N at 298K

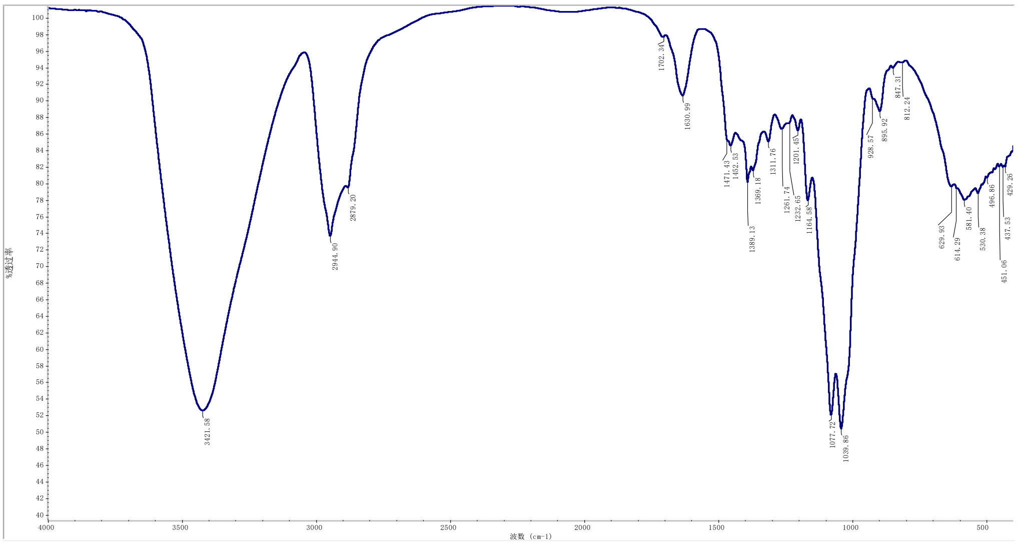

Figure S25. IR spectrum of **3**

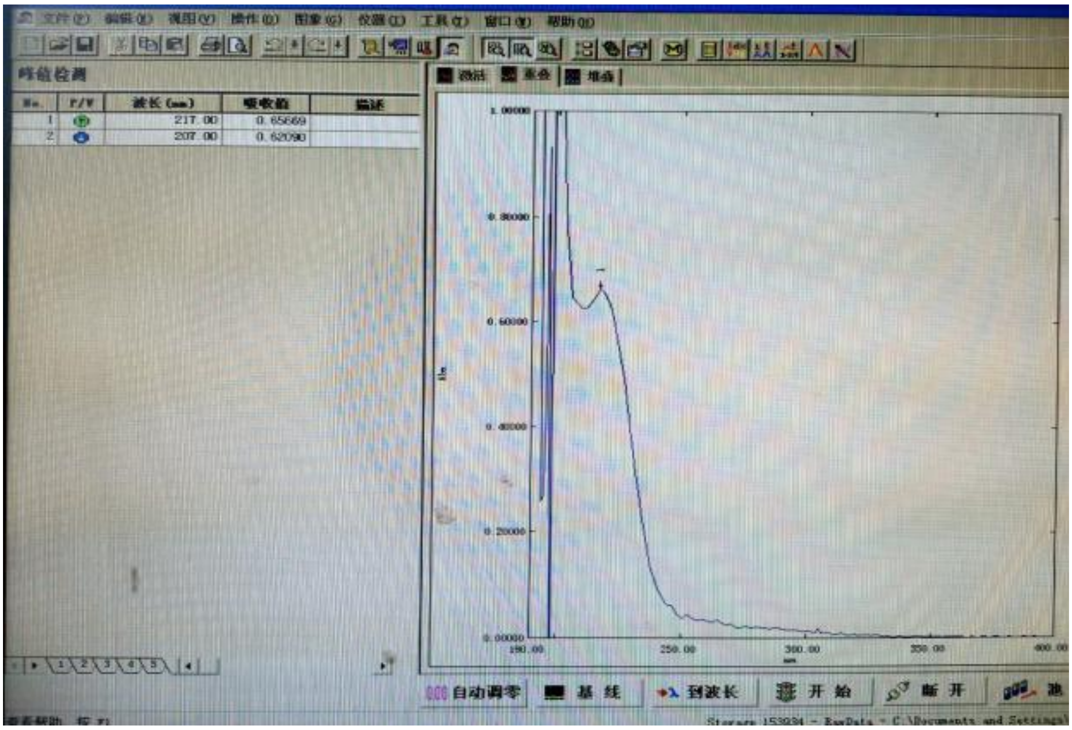

Figure S26. UV spectrum of **3**

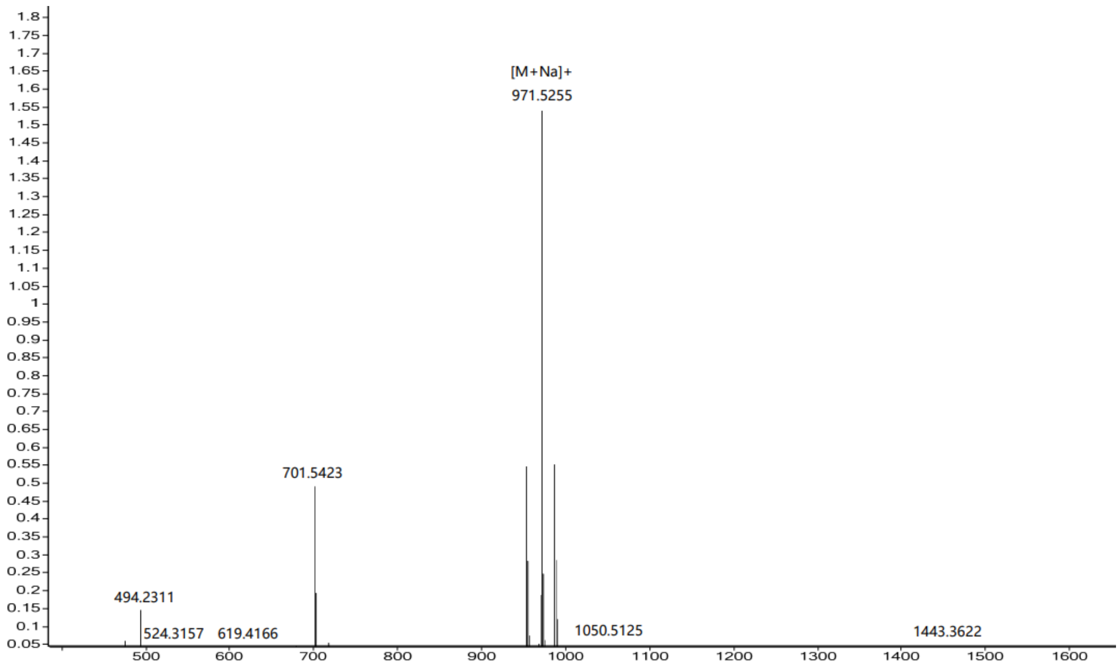

Figure S27. HRESI spectrum of **3**

Rudolph Research Analytical

This sample was measured on an Autopol VI, Serial #91058
Manufactured by Rudolph Research Analytical, Hackettstown, NJ, USA.

Measurement Date : Thursday, 27-FEB-2020

Set Temperature : OFF

Time Delay : Disabled

Delay between Measurement : Disabled

n	Average	Std.Dev.	% RSD	Maximum	Minimum
5	7.89	0.25	3.16	8.33	7.78

S.No	Sample ID	Time	Result	Scale	OR °Arc	WLG.nm	Lg.mm	Conc.g/100ml	Temp.
1	GY-19	02:43:12 PM	7.78	SR	0.014	589	100.00	0.180	23.4
2	GY-19	02:43:17 PM	7.78	SR	0.014	589	100.00	0.180	23.4
3	GY-19	02:43:23 PM	7.78	SR	0.014	589	100.00	0.180	23.4
4	GY-19	02:43:29 PM	8.33	SR	0.015	589	100.00	0.180	23.4
5	GY-19	02:43:34 PM	7.78	SR	0.014	589	100.00	0.180	23.4

Figure S28. $[\alpha]_{\mathrm{D}}^{23}$ of **3**

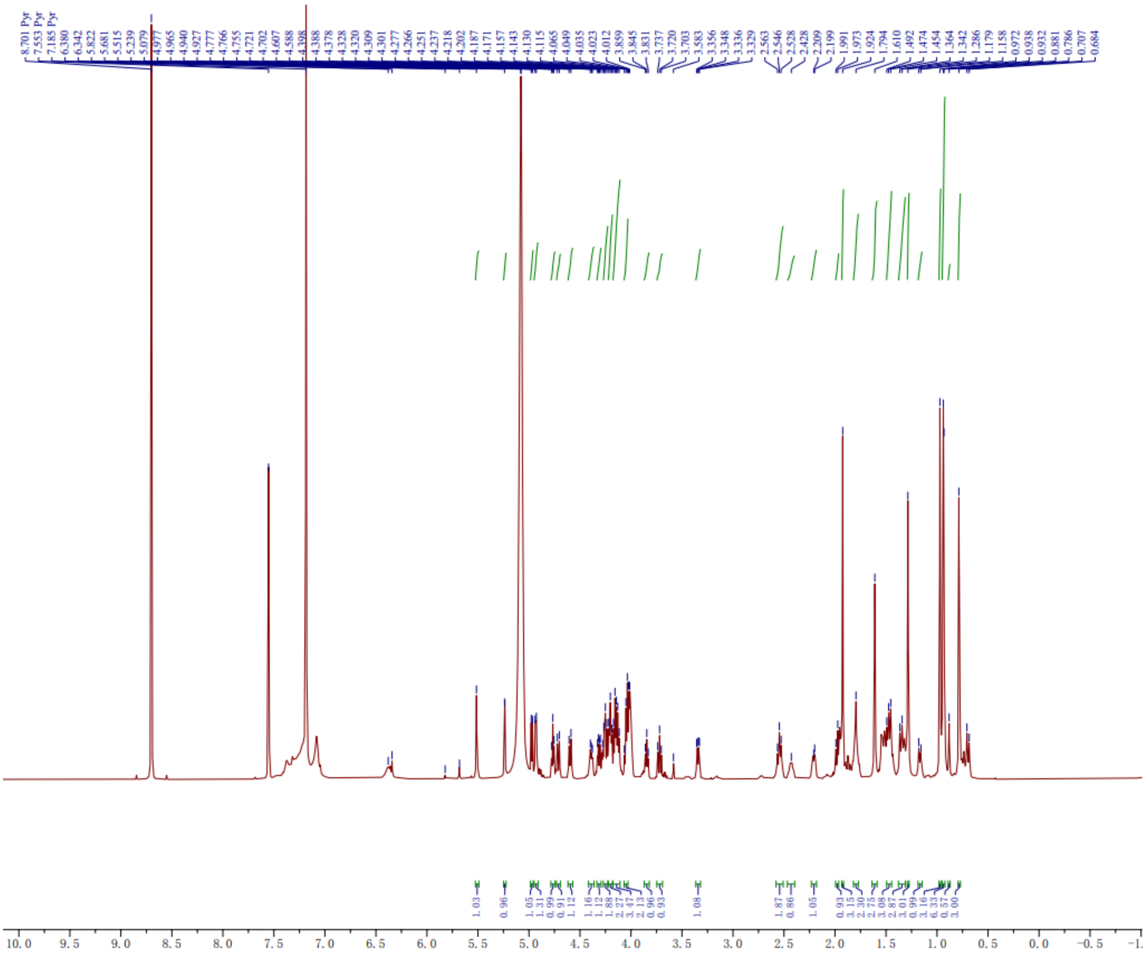

Figure S29. ^{1}H-NMR spectrum of **3** in C_5D_5N at 298K (600 MHz)

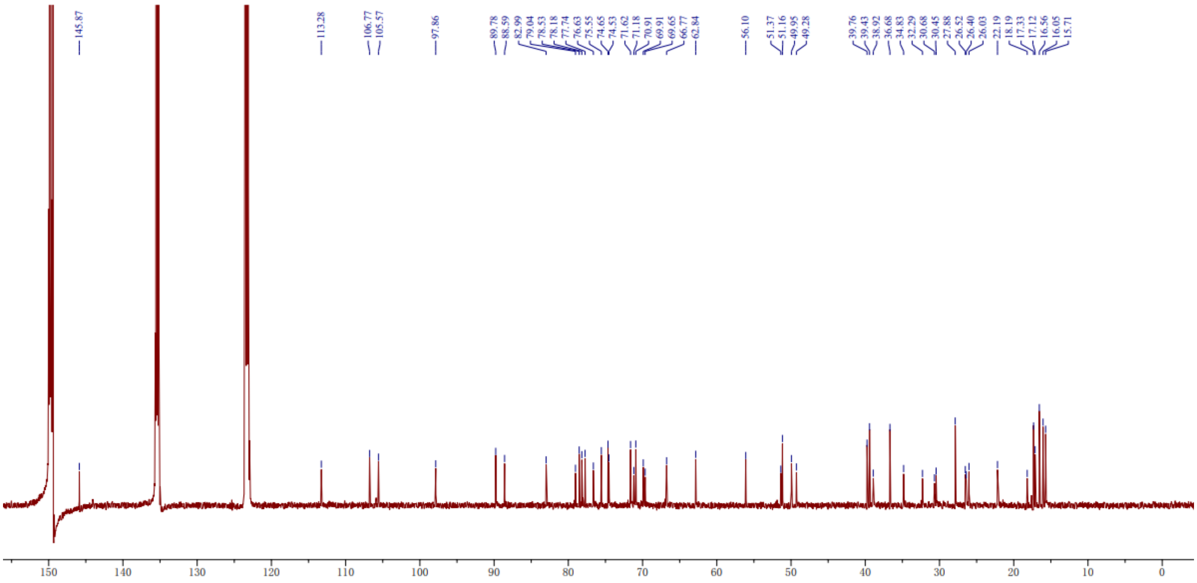

Figure S30. ^{13}C-NMR spectrum of **3** in C_5D_5N at 298K (150 MHz)

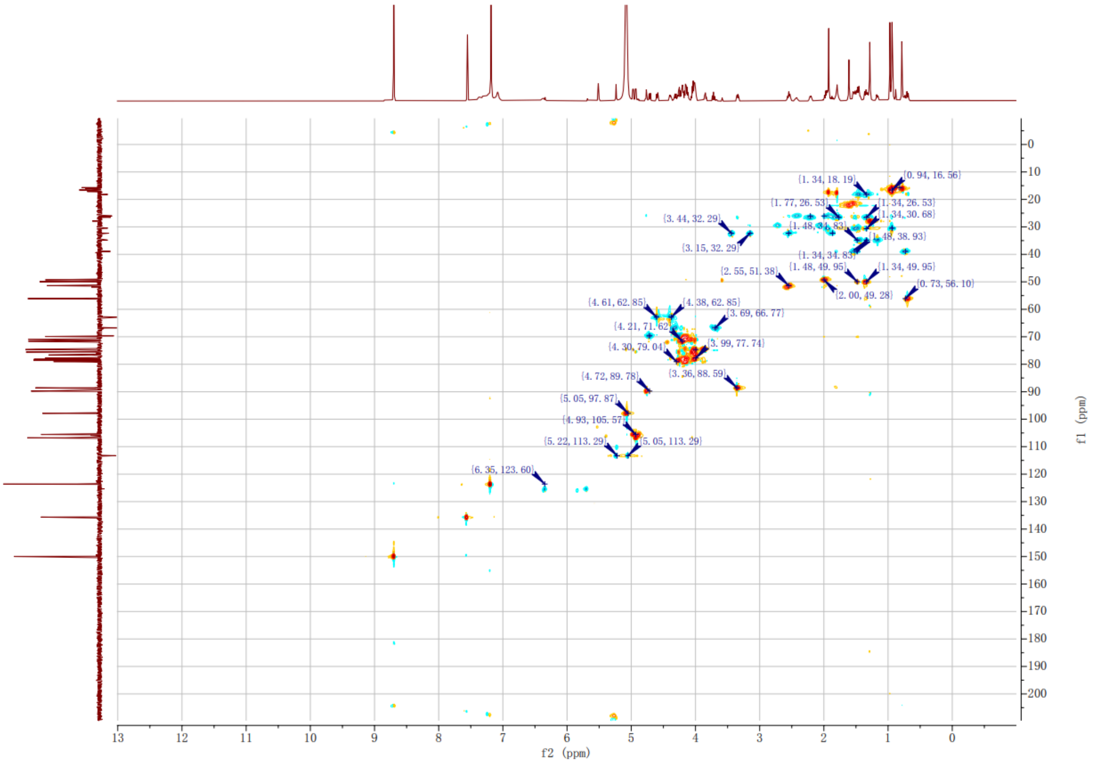

Figure S31. HSQC spectrum of **3** in C_5D_5N at 298K

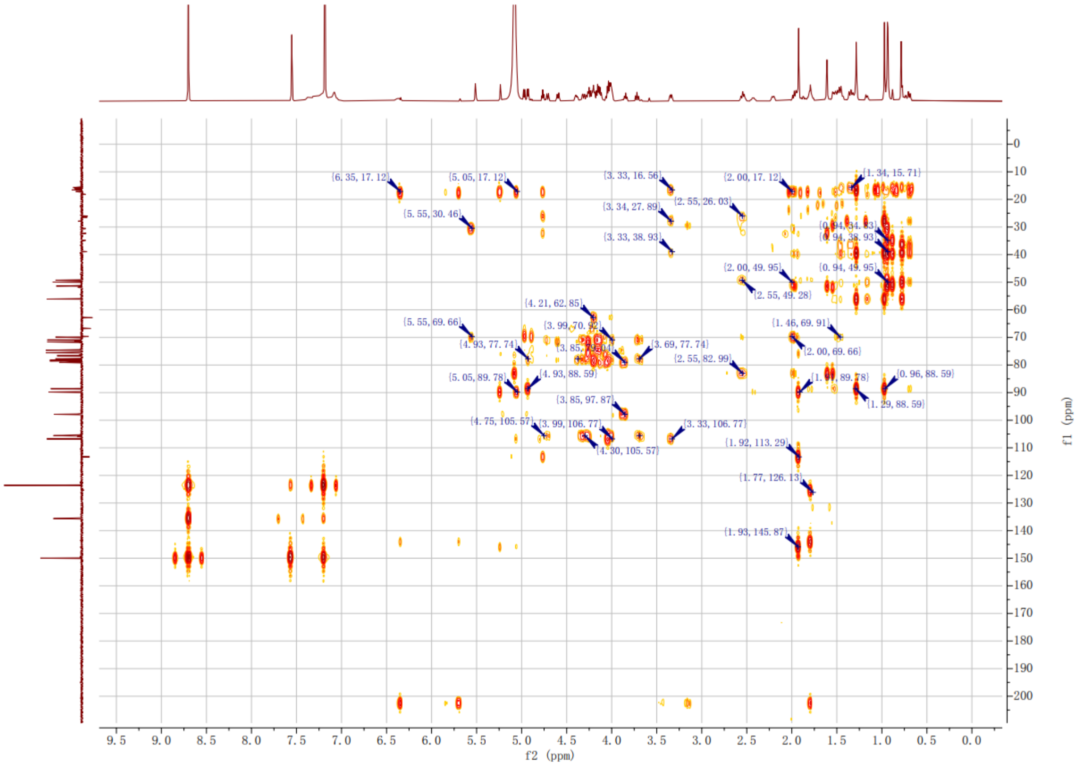

Figure S32. HMBC spectrum of **3** in C_5D_5N at 298K

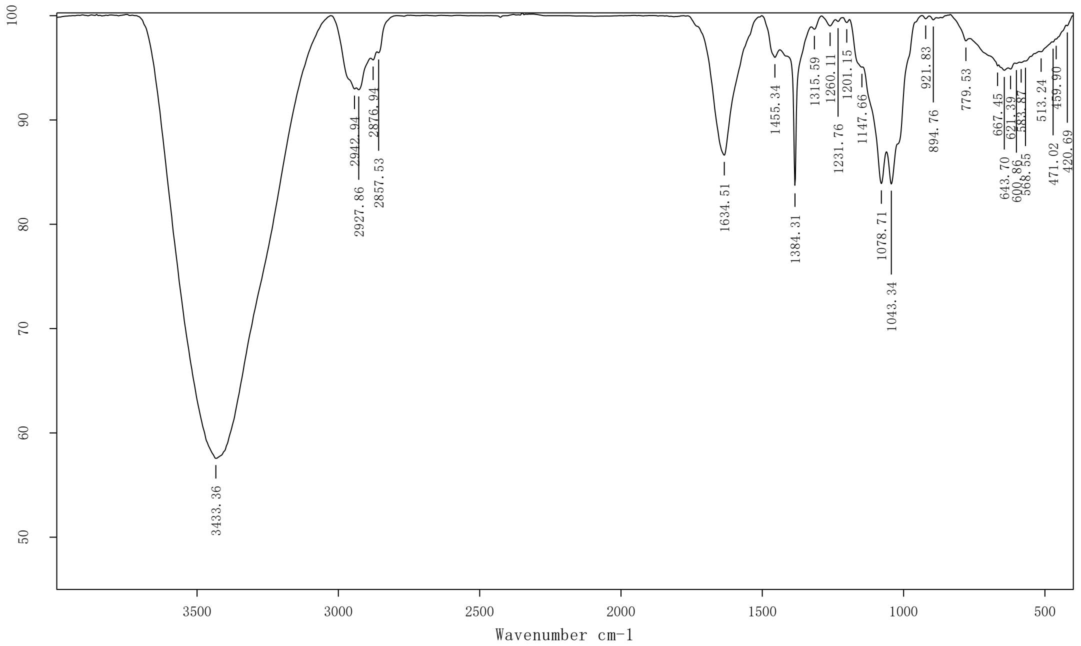

Figure S33. IR spectrum of **4**

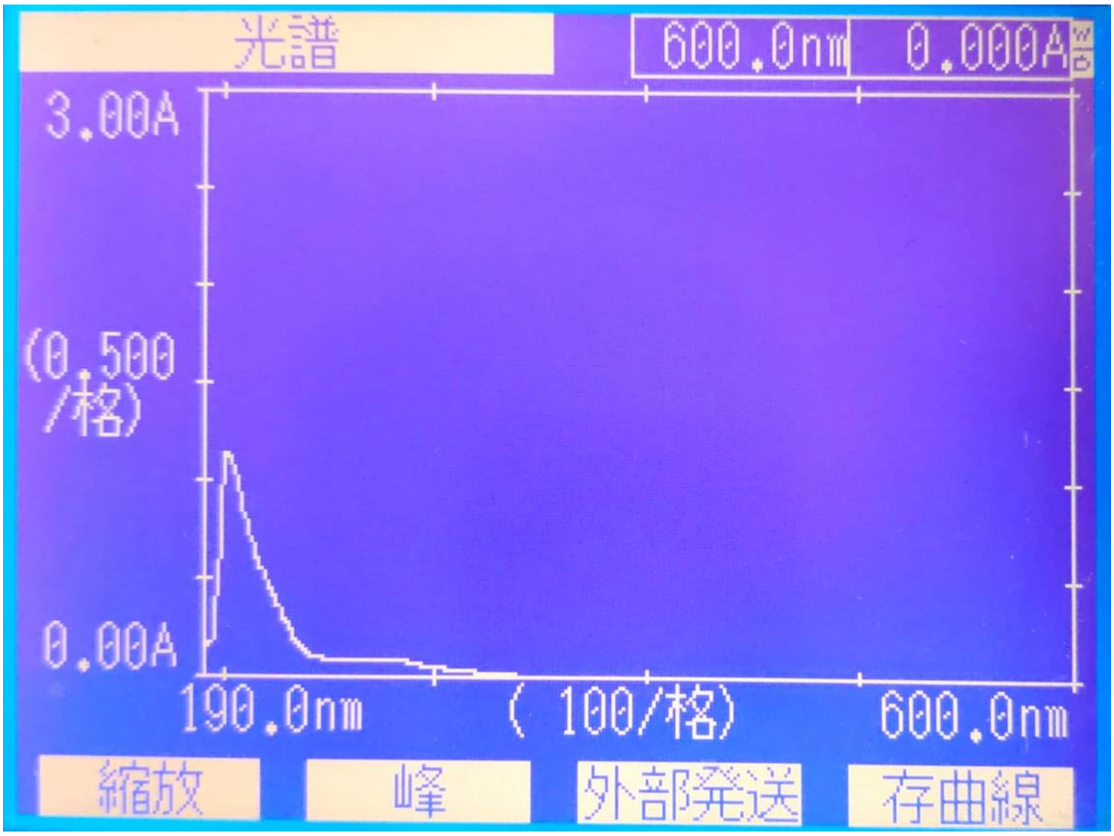

Figure S34. UV spectrum of **4**

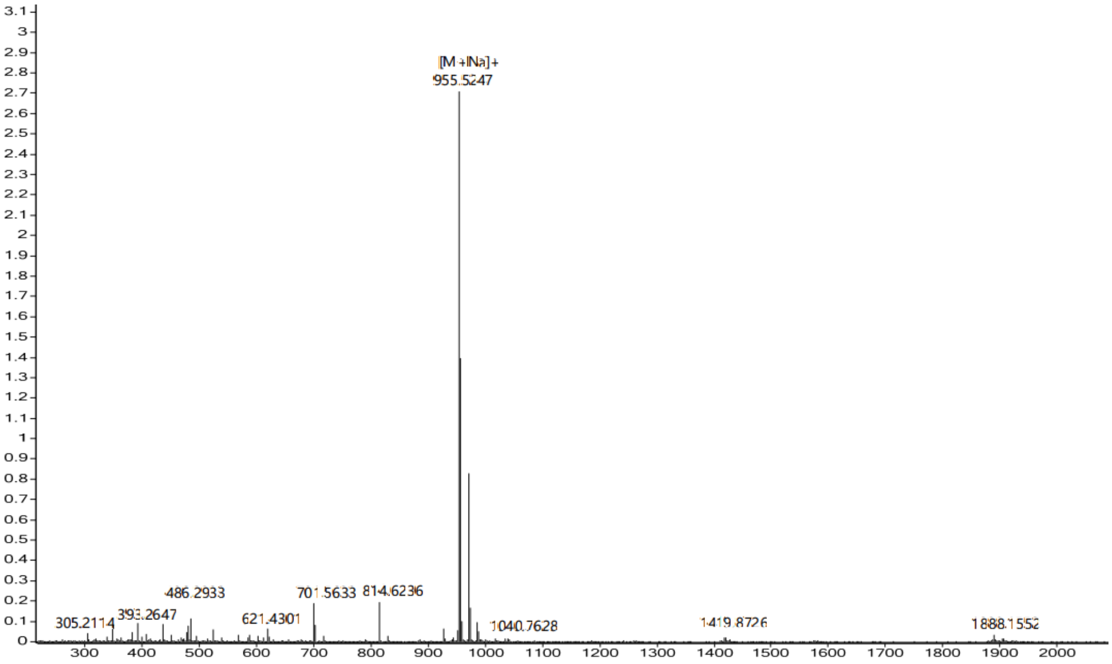

Figure S35. HRESI of **4**

Rudolph Research Analytical

This sample was measured on an Autopol VI, Serial #91058
Manufactured by Rudolph Research Analytical, Hackettstown, NJ, USA.

Measurement Date : Thursday, 14-OCT-2021

Set Temperature : OFF

Time Delay : Disabled

Delay between Measurement : Disabled

n	Average	Std.Dev.	% RSD	Maximum	Minimum
5	19.20	1.10	5.72	20.00	18.00

S.No	Sample ID	Time	Result	Scale	OR °Arc	WLG.nm	Lg.mm	Conc.g/100ml	Temp.
1	wxy-8-7	05:15:51 PM	20.00	SR	0.010	589	100.00	0.050	22.9
2	wxy-8-7	05:15:57 PM	20.00	SR	0.010	589	100.00	0.050	22.9
3	wxy-8-7	05:16:04 PM	20.00	SR	0.010	589	100.00	0.050	22.9
4	wxy-8-7	05:16:14 PM	18.00	SR	0.009	589	100.00	0.050	22.9
5	wxy-8-7	05:16:20 PM	18.00	SR	0.009	589	100.00	0.050	22.9

Figure S36. $[\alpha]_{D}^{23}$ of **4**

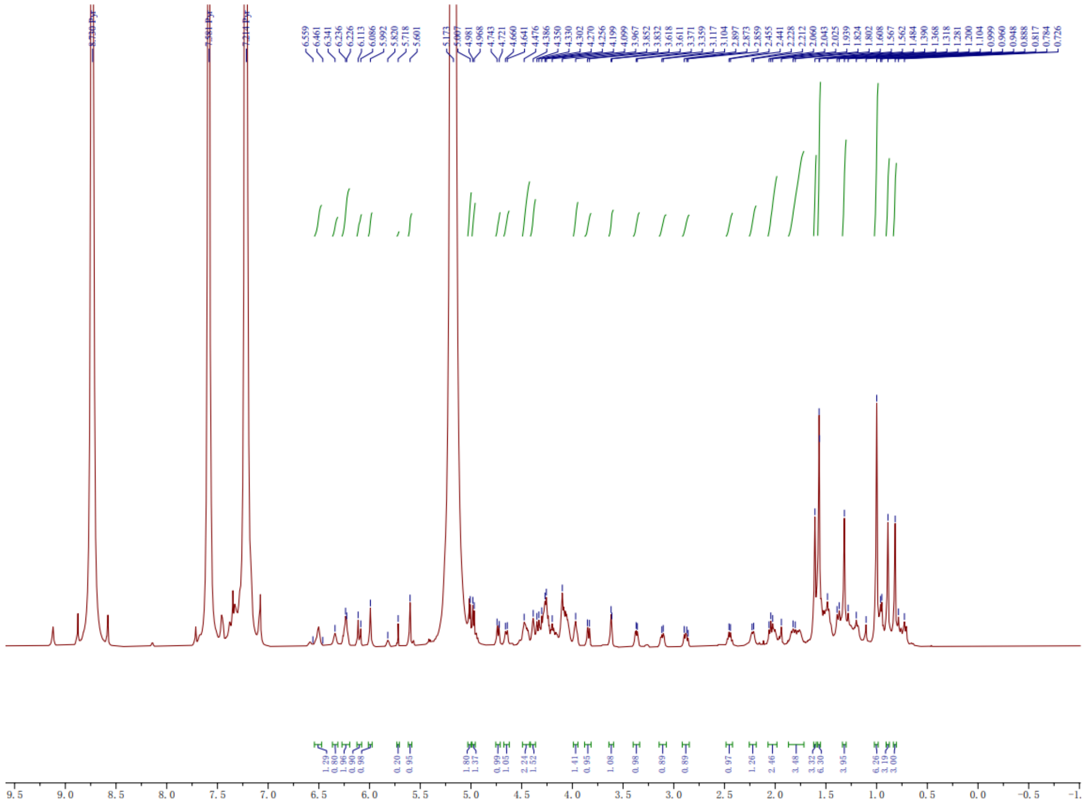

Figure S37. ^{1}H-NMR spectrum of **4** in C_5D_5N at 298K (600 MHz)

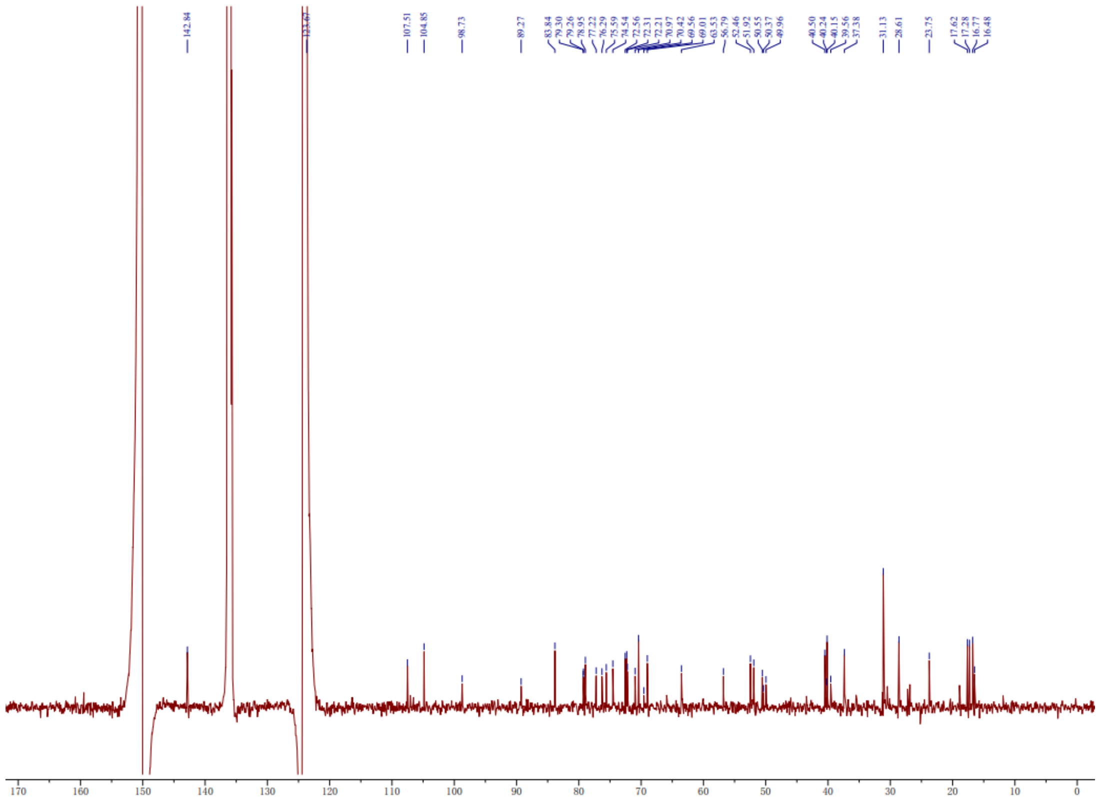

Figure S38. ^{1}H-NMR spectrum of **4** in C_5D_5N at 298K (150 MHz)

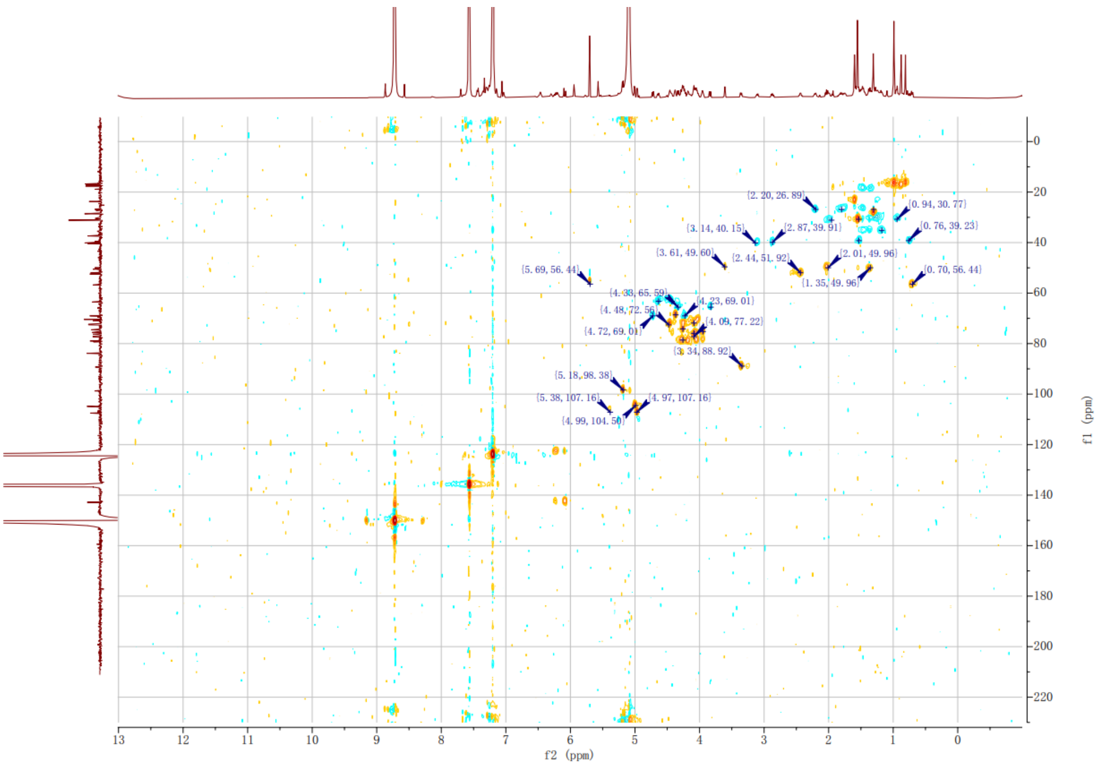

Figure S39. ¹H-NMR spectrum of **4** in C_5D_5N at 298K

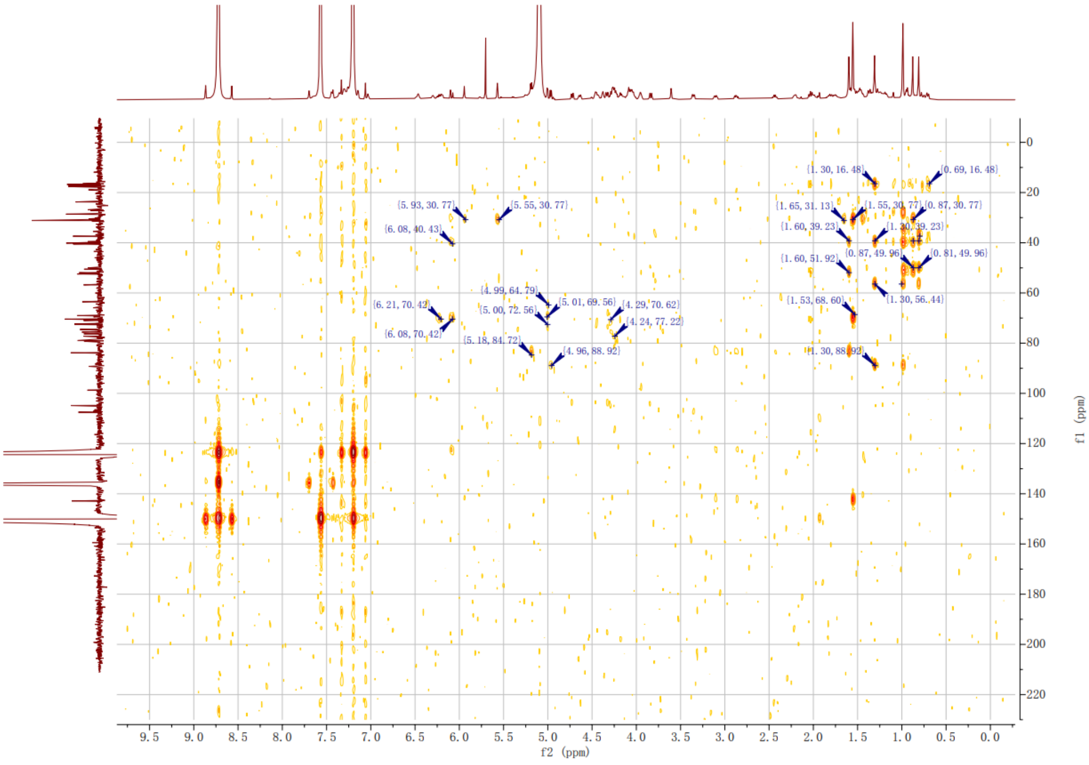

Figure S40. ¹H-NMR spectrum of **4** in C_5D_5N at 298K

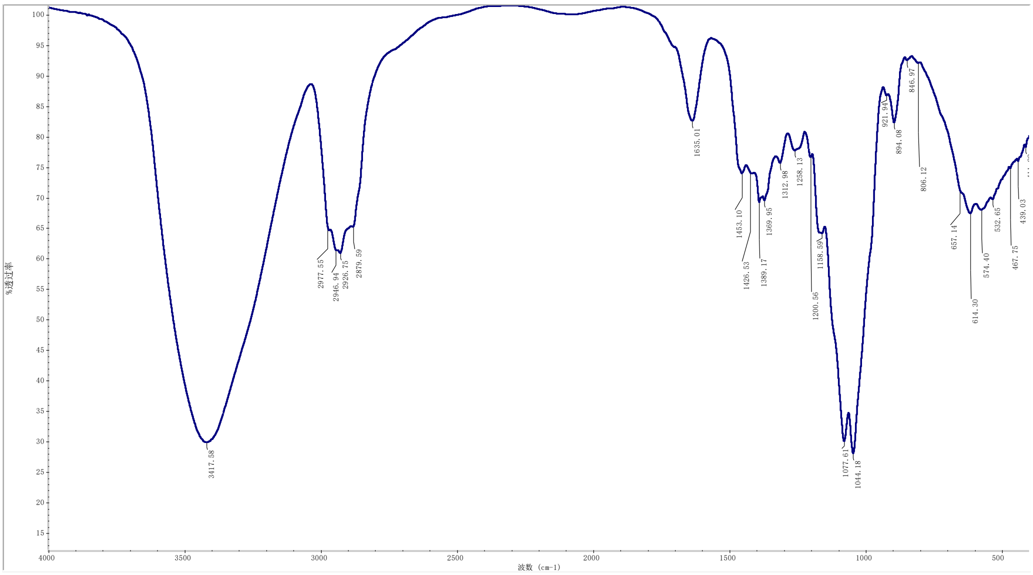

Figure S41. IR spectrum of **5**

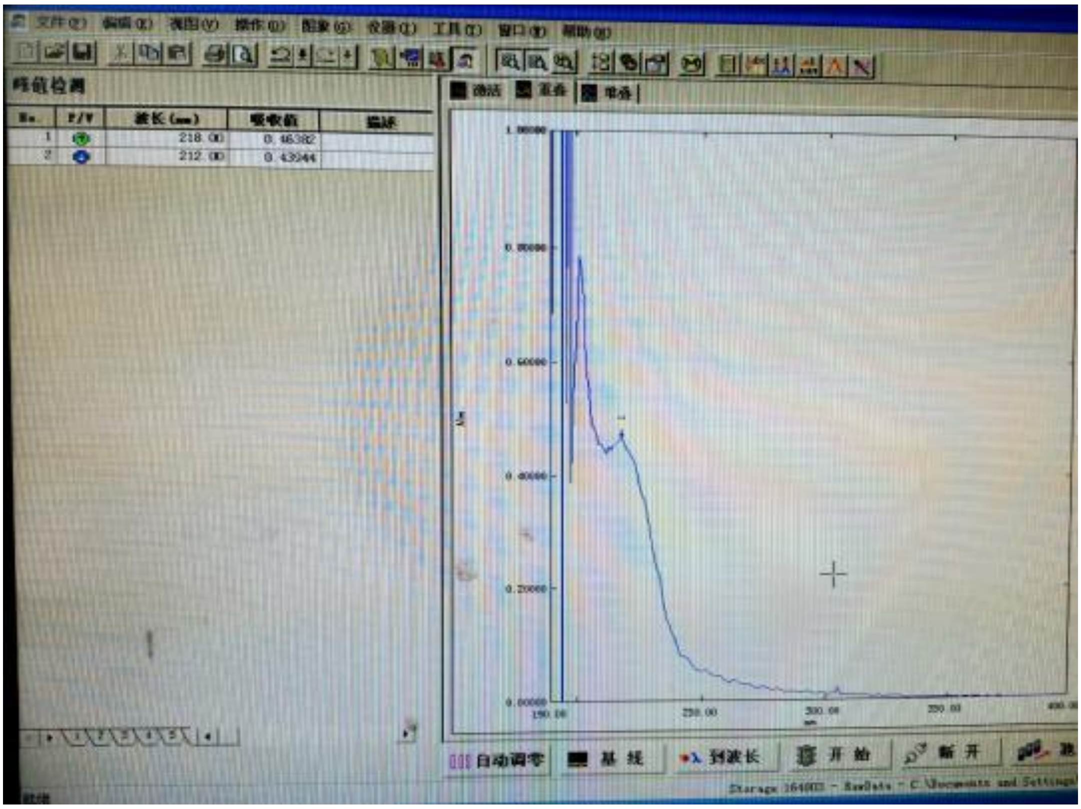

Figure S42. UV spectrum of **5**

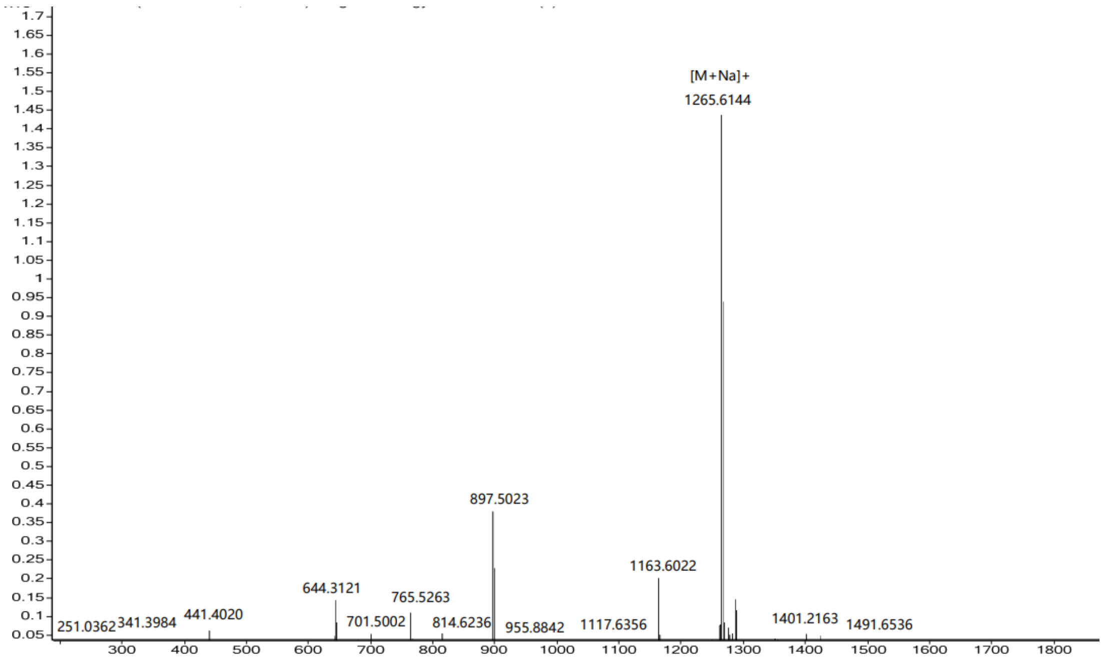

Figure S43. HRESI of **5**

Rudolph Research Analytical

This sample was measured on an Autopol VI, Serial #91058
Manufactured by Rudolph Research Analytical, Hackettstown, NJ, USA.

Measurement Date : Thursday, 27-FEB-2020

Set Temperature : OFF

Time Delay : Disabled

Delay between Measurement : Disabled

n	Average	Std.Dev.	% RSD	Maximum	Minimum
5	10.67	0.25	2.34	11.11	10.56

S.No	Sample ID	Time	Result	Scale	OR °Arc	WLG.nm	Lg.mm	Conc.g/100ml	Temp.
1	GY-42	03:19:10 PM	10.56	SR	0.019	589	100.00	0.180	22.9
2	GY-42	03:19:16 PM	10.56	SR	0.019	589	100.00	0.180	22.9
3	GY-42	03:19:22 PM	10.56	SR	0.019	589	100.00	0.180	22.9
4	GY-42	03:19:28 PM	11.11	SR	0.020	589	100.00	0.180	22.9
5	GY-42	03:19:33 PM	10.56	SR	0.019	589	100.00	0.180	22.9

Figure S44. $[\alpha]_{\mathrm{D}}^{23}$ of **5**

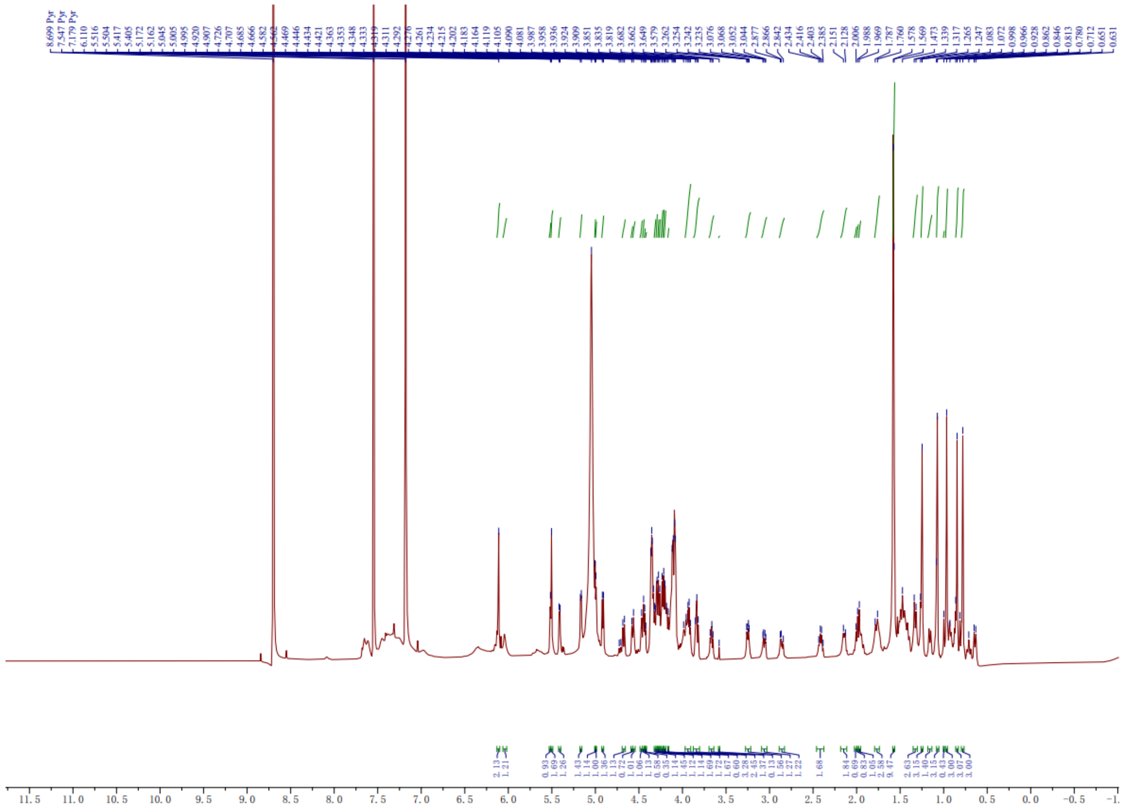

Figure S45. ^{1}H-NMR spectrum of **5** in C_5D_5N at 298K (600 MHz)

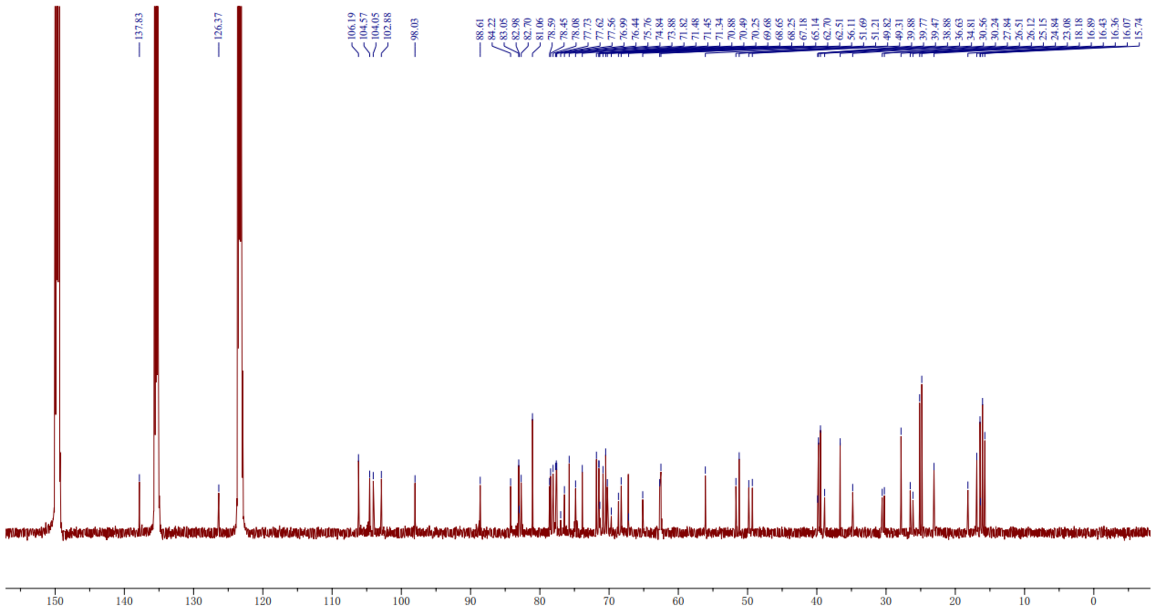

Figure 46. ^{13}C-NMR spectrum of **5** in C_5D_5N at 298K (150 MHz)

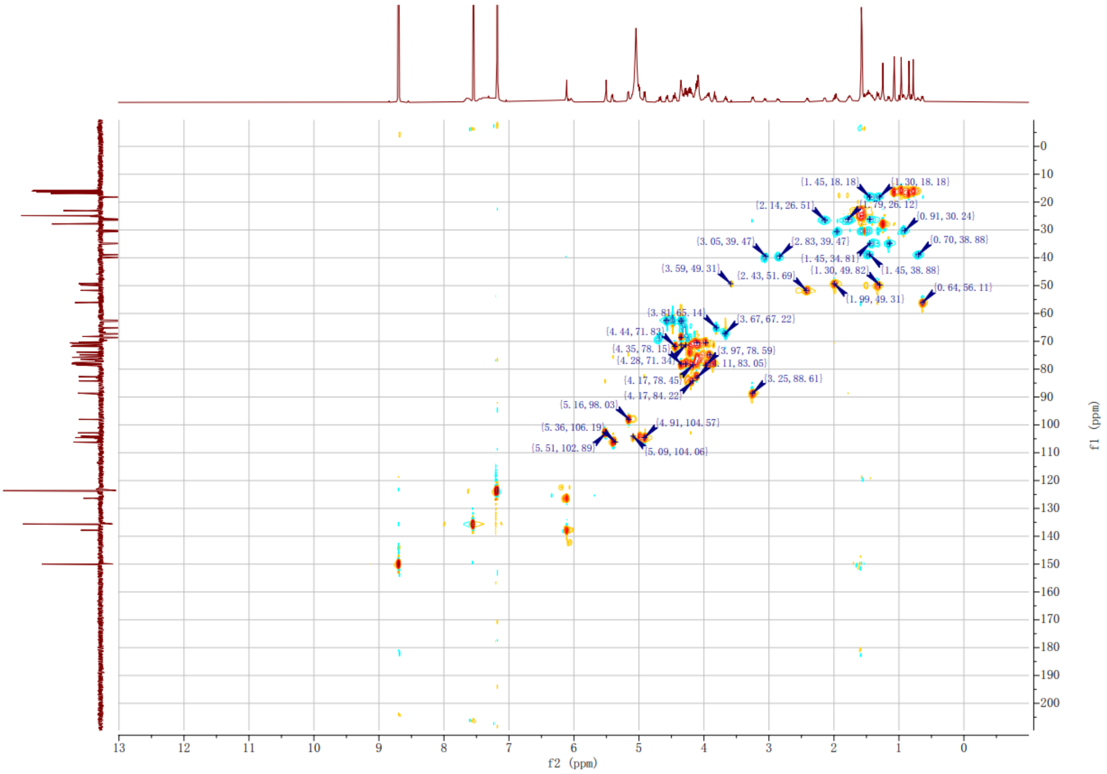

Figure S47. HSQC spectrum of **5** in C_5D_5N at 298K

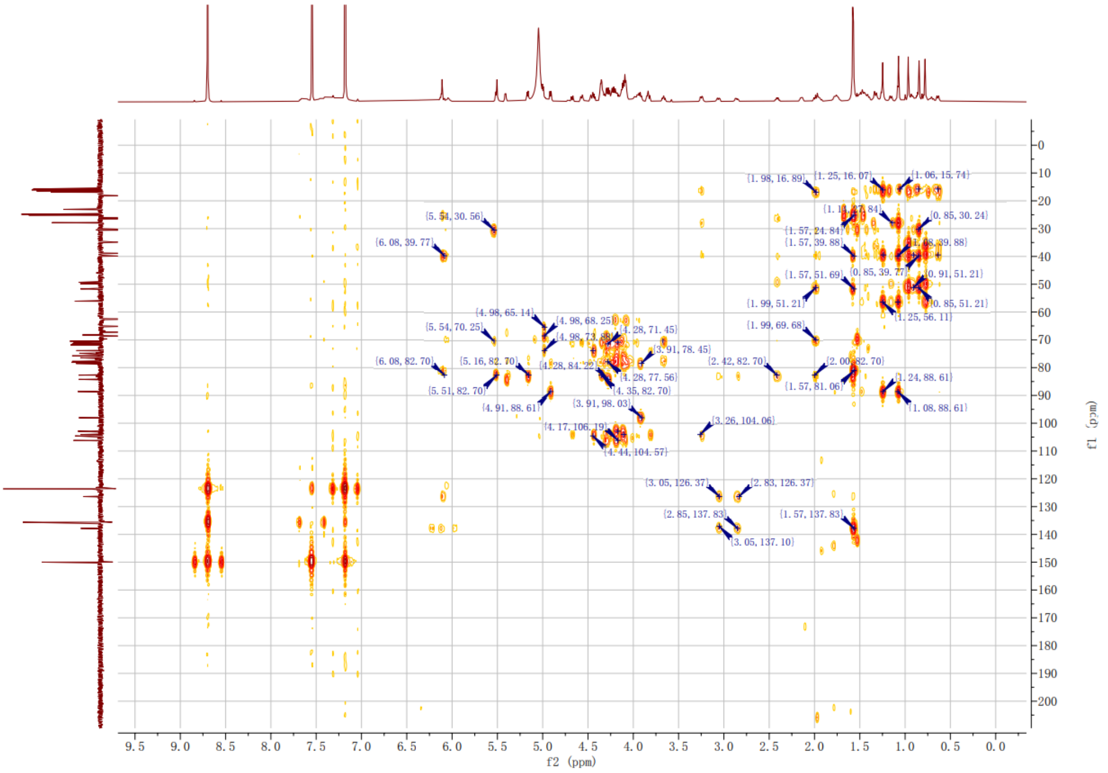

Figure S48. HMBC spectrum of **5** in C_5D_5N at 298K

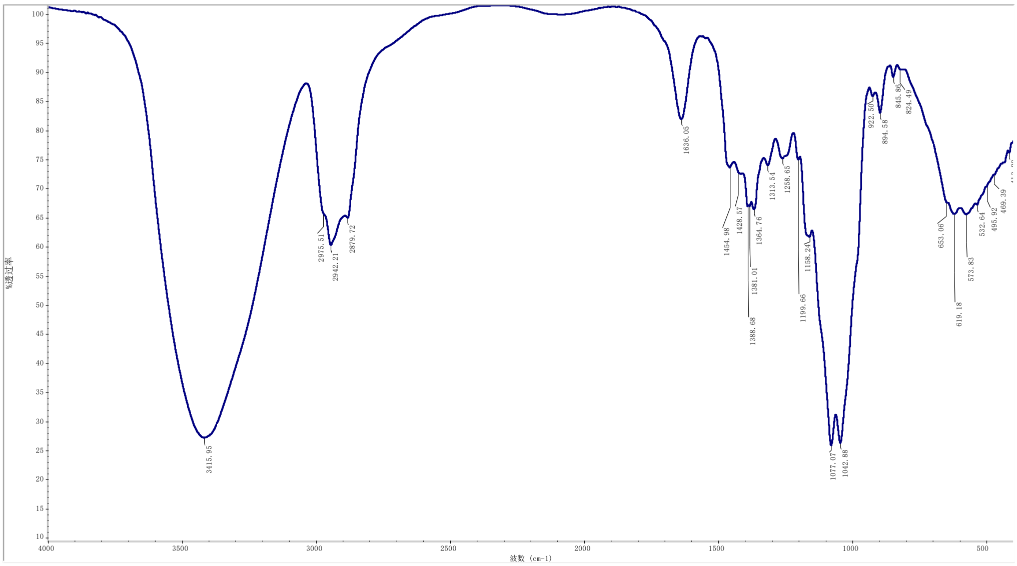

Figure S49. IR spectrum of **6**

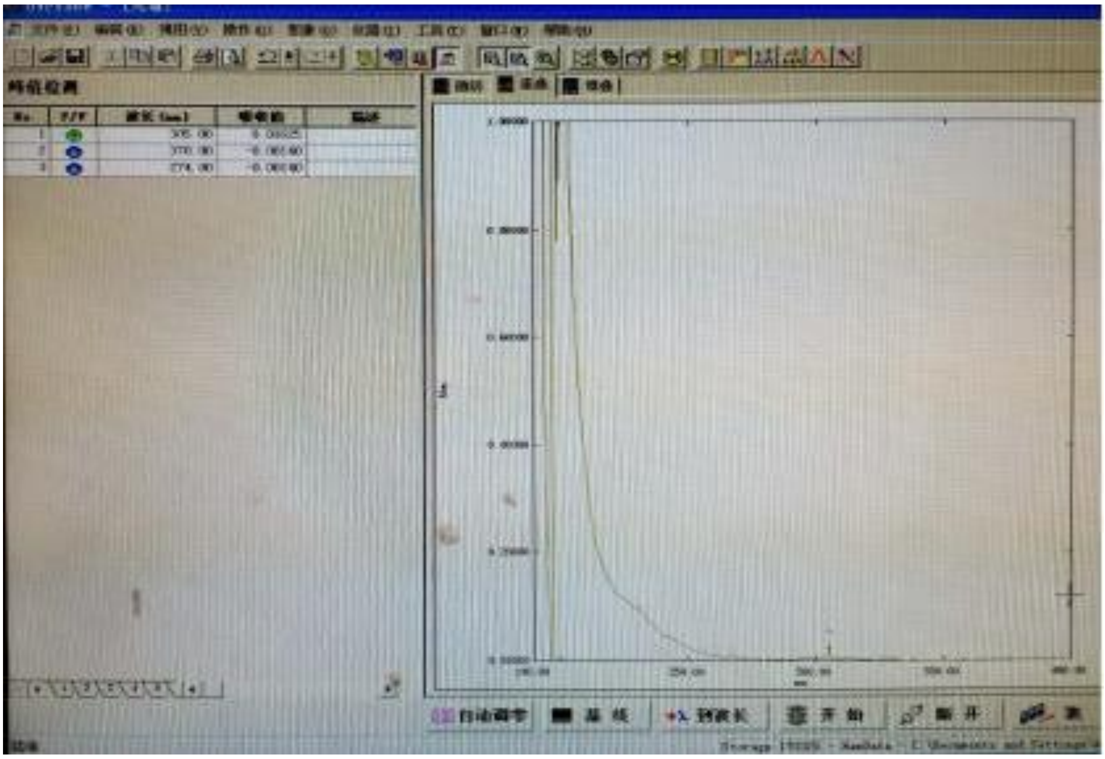

Figure S50. UV spectrum of **6**

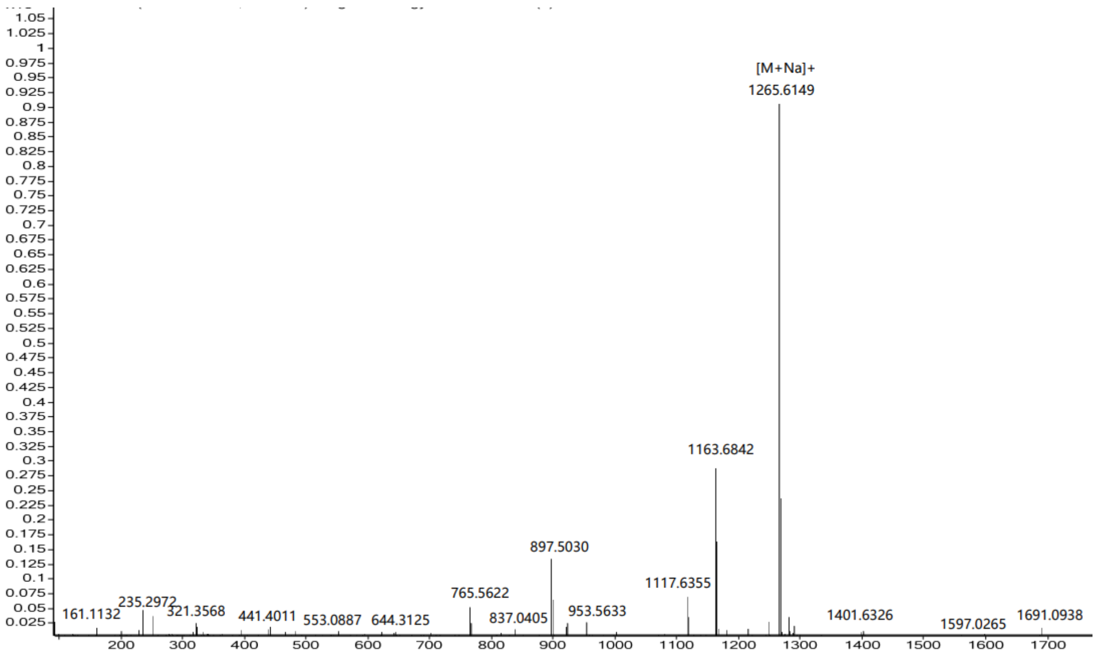

Figure S51. HRESI of **6**

Rudolph Research Analytical

This sample was measured on an Autopol VI, Serial #91058
Manufactured by Rudolph Research Analytical, Hackettstown, NJ, USA.

Measurement Date : Thursday, 27-FEB-2020

Set Temperature : OFF

Time Delay : Disabled

Delay between Measurement : Disabled

n	Average	Std.Dev.	% RSD	Maximum	Minimum
5	7.45	0.19	2.55	7.59	7.24

S.No	Sample ID	Time	Result	Scale	OR °Arc	WLG.nm	Lg.mm	Conc.g/100ml	Temp.
1	GY-41	03:50:27 PM	7.59	SR	0.022	589	100.00	0.290	22.7
2	GY-41	03:50:33 PM	7.24	SR	0.021	589	100.00	0.290	22.7
3	GY-41	03:50:39 PM	7.24	SR	0.021	589	100.00	0.290	22.7
4	GY-41	03:50:45 PM	7.59	SR	0.022	589	100.00	0.290	22.7
5	GY-41	03:50:50 PM	7.59	SR	0.022	589	100.00	0.290	22.7

Figure S52. $[\alpha]_{\mathrm{D}}^{23}$ of **6**

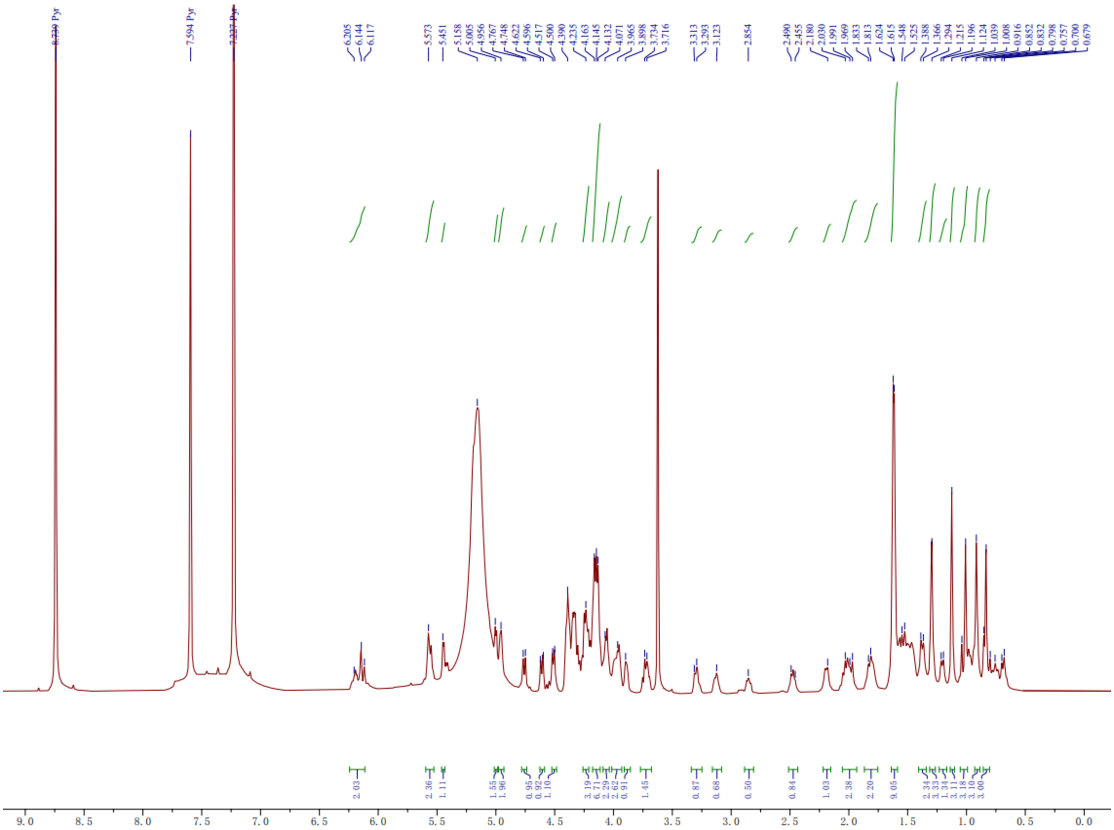

Figure S53. ^{1}H-NMR spectrum of **6** in C_5D_5N at 298K (600 MHz)

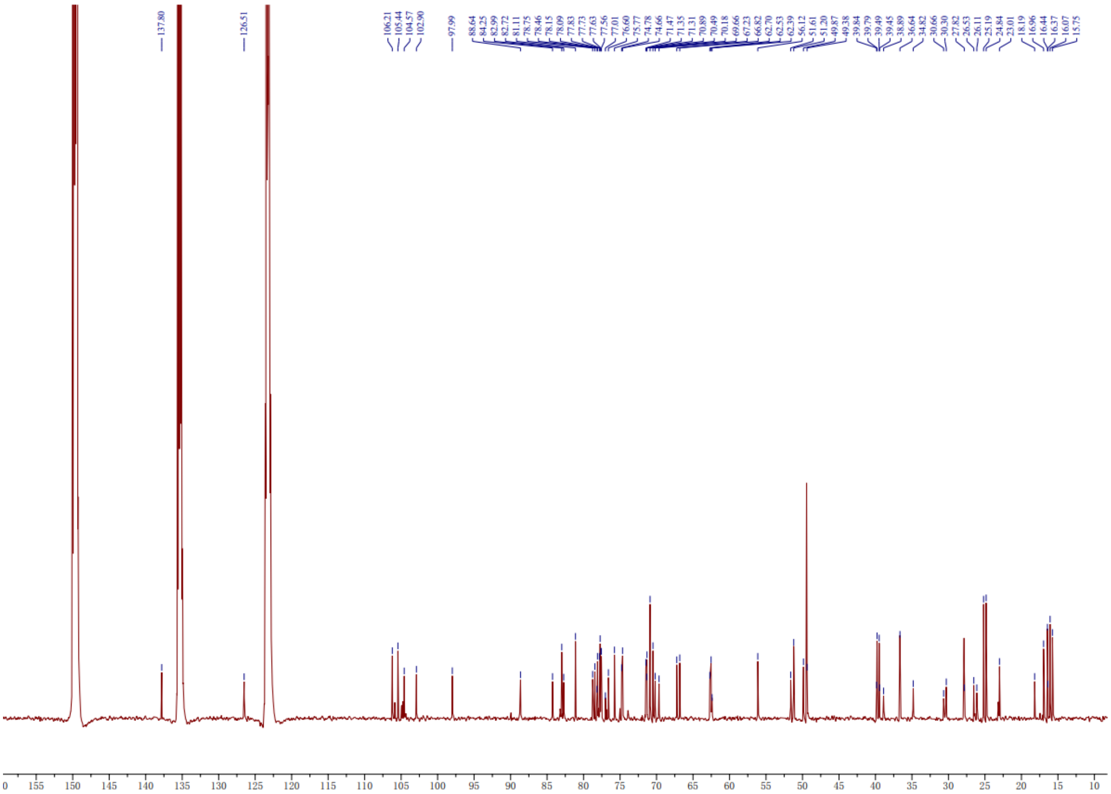

Figure S54. ^{13}C-NMR spectrum of **6** in C_5D_5N at 298K (150 MHz)

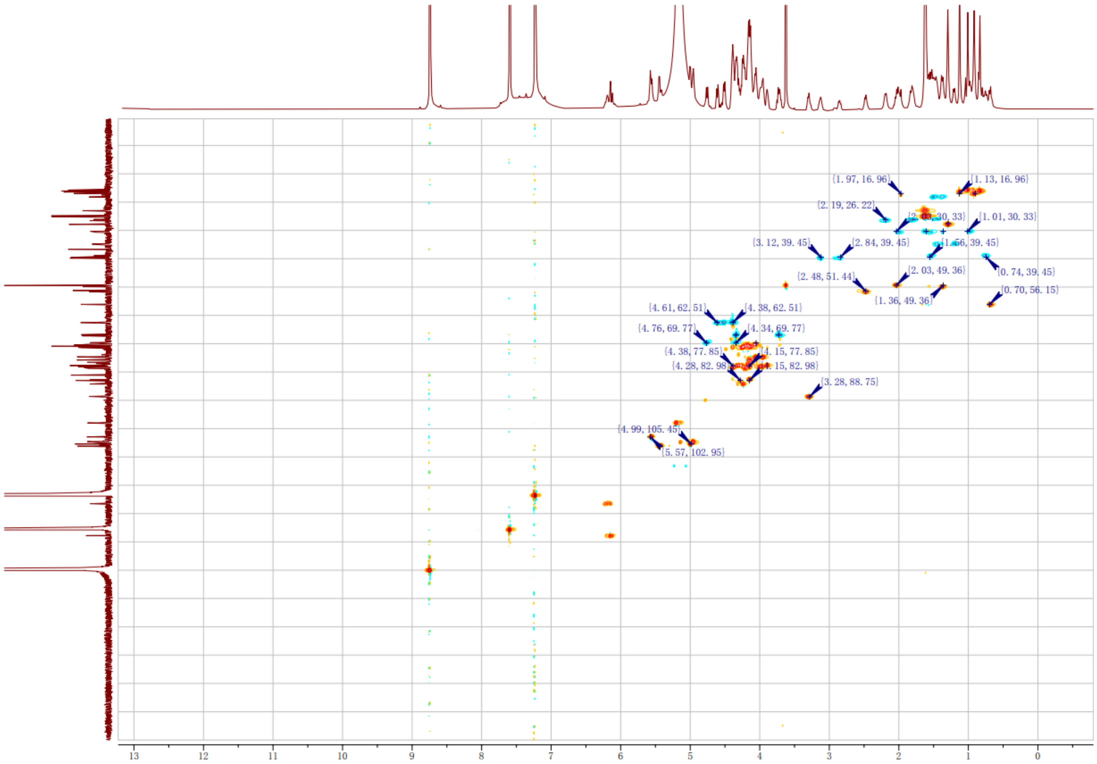

Figure S55. HSQC spectrum of **6** in C_5D_5N at 298K

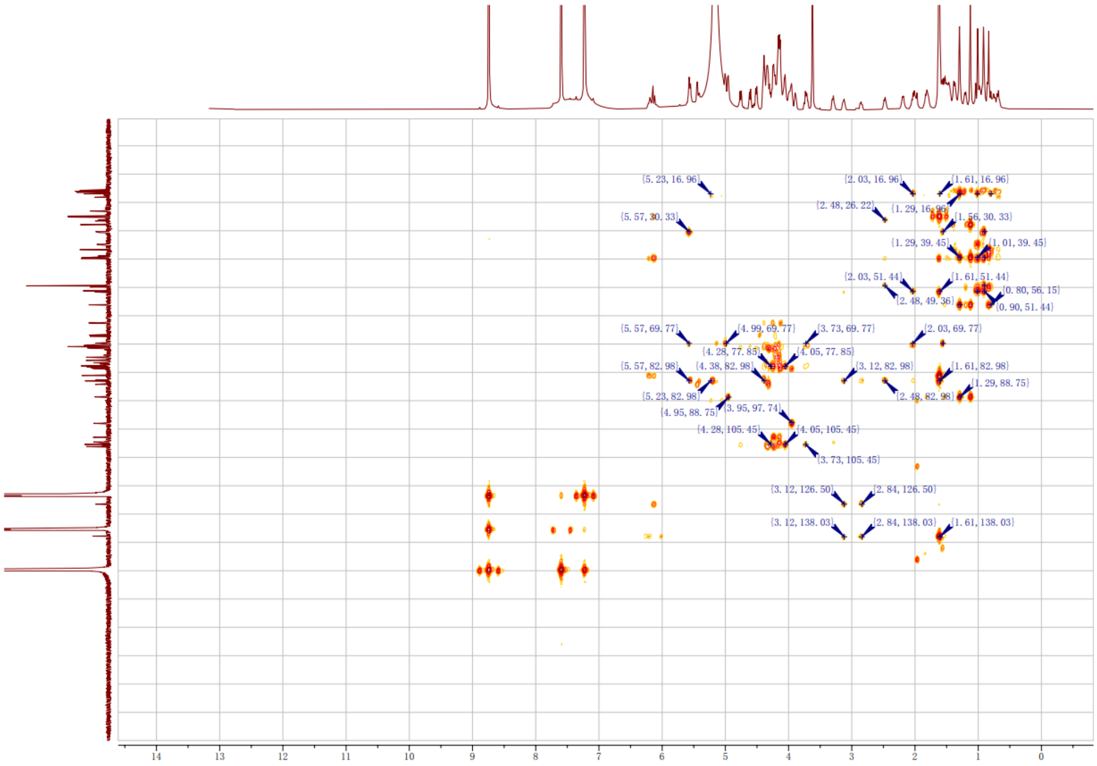

Figure S56. HMBC spectrum of **6** in C_5D_5N at 298K

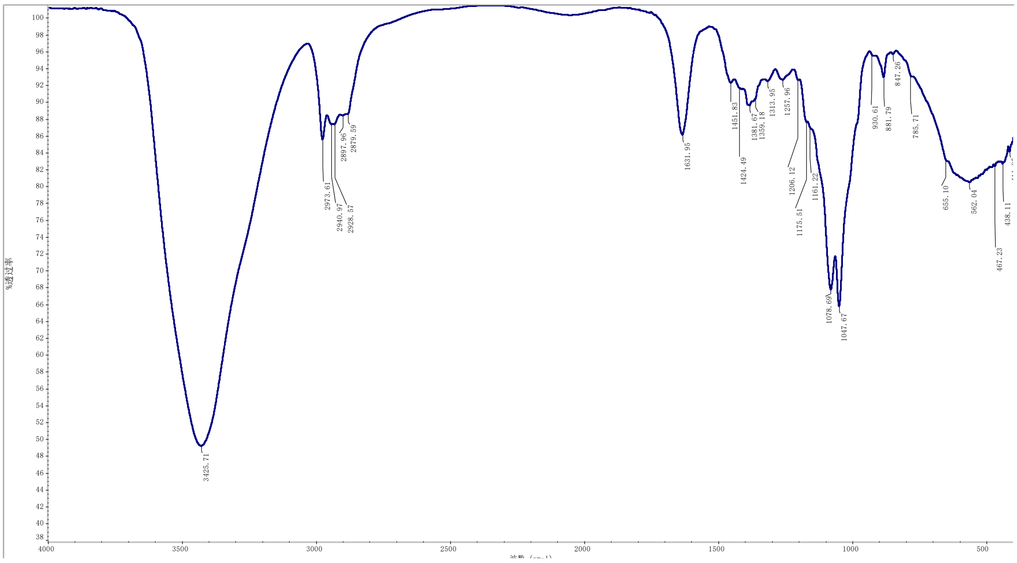

Figure S57. IR spectrum of **7**

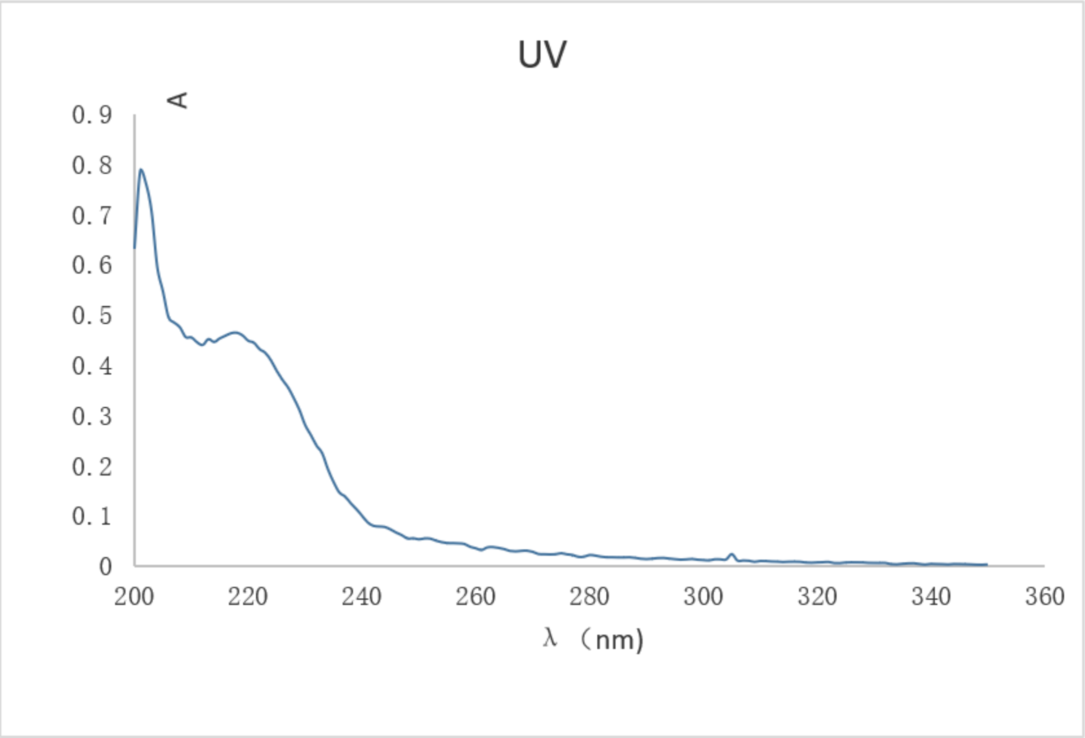

Figure S58. UV spectrum of **7**

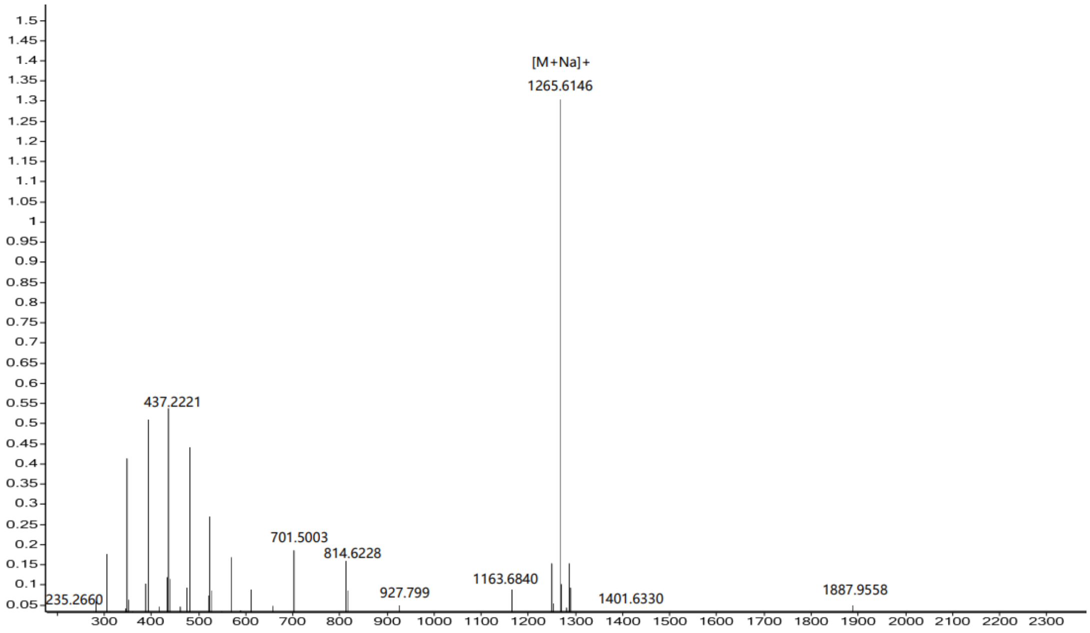

Figure S59. HRESI of **7**

<u>**Rudolph Research Analytical**</u>

This sample was measured on an Autopol VI, Serial #91058
Manufactured by Rudolph Research Analytical, Hackettstown, NJ, USA.

Measurement Date : Thursday, 27-FEB-2020

Set Temperature : OFF

Time Delay : Disabled

Delay between Measurement : Disabled

n	Average	Std.Dev.	% RSD	Maximum	Minimum
5	4.40	0.60	13.63	5.33	4.00

S.No	Sample ID	Time	Result	Scale	OR °Arc	WLG.nm	Lg.mm	Conc.g/100ml	Temp.
1	GY-39	03:06:11 PM	5.33	SR	0.008	589	100.00	0.150	23.8
2	GY-39	03:06:17 PM	4.67	SR	0.007	589	100.00	0.150	23.8
3	GY-39	03:06:23 PM	4.00	SR	0.006	589	100.00	0.150	23.8
4	GY-39	03:06:28 PM	4.00	SR	0.006	589	100.00	0.150	23.8
5	GY-39	03:06:34 PM	4.00	SR	0.006	589	100.00	0.150	23.8

Figure S60. $[\alpha]_{\mathrm{D}}^{24}$ of **7**

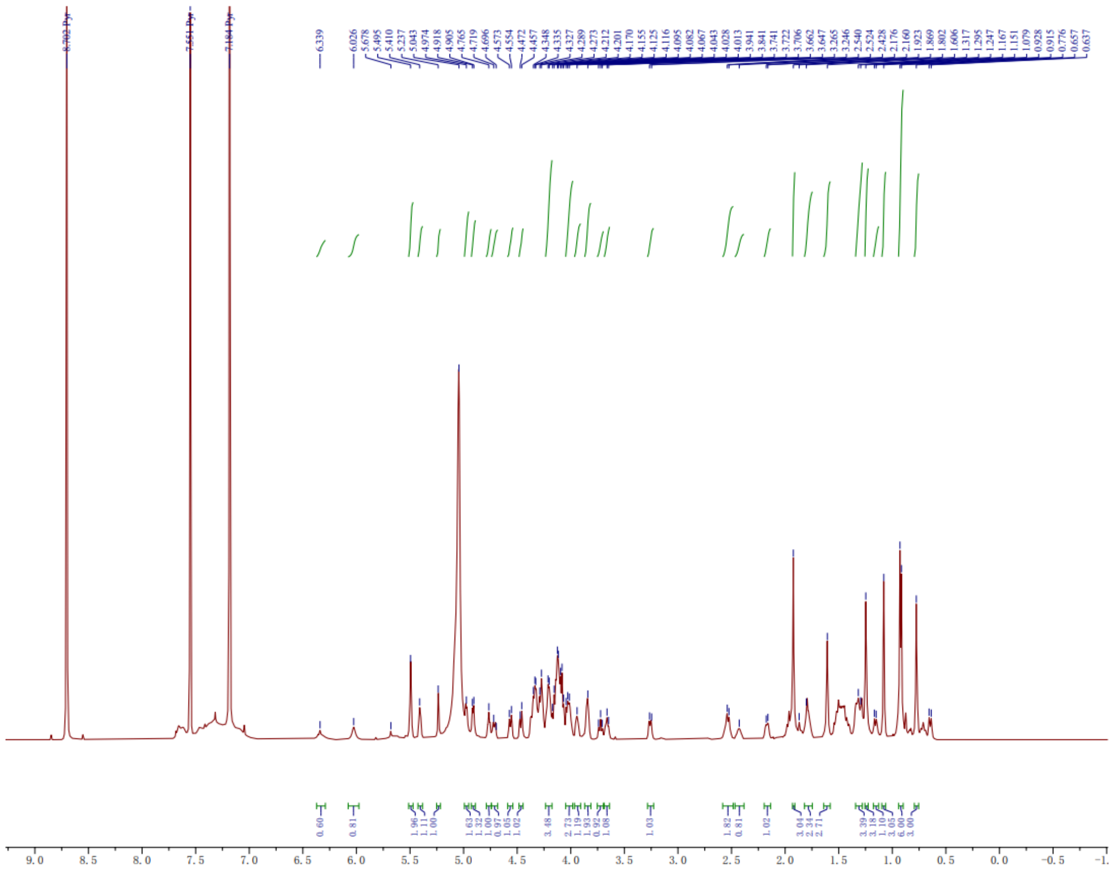

Figure S61. ^{1}H-NMR spectrum of **7** in C_5D_5N at 298K (600 MHz)

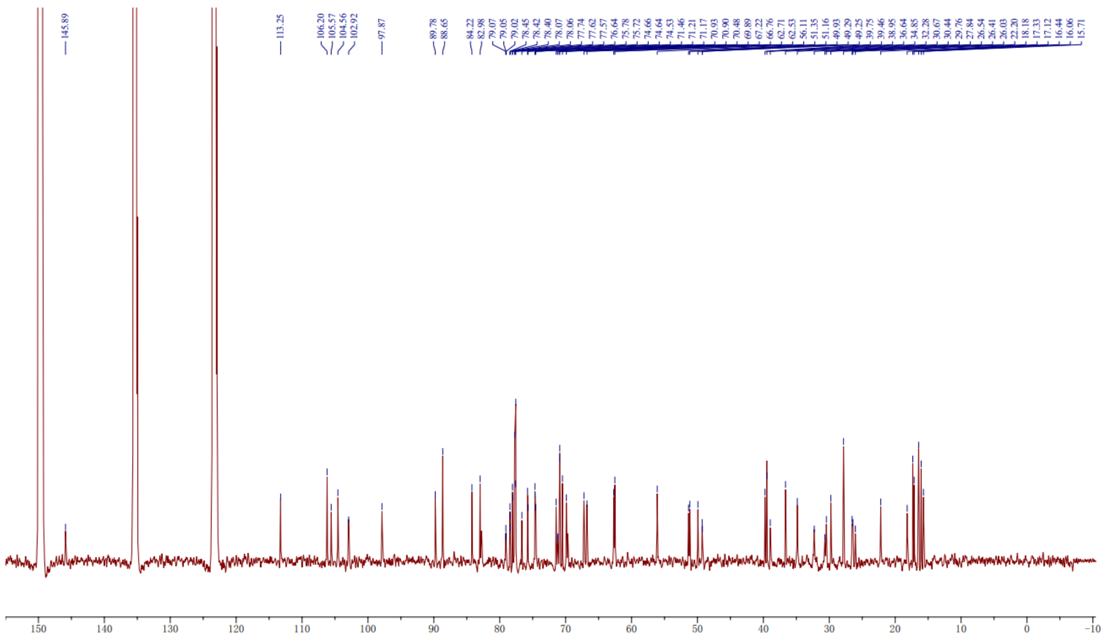

Figure S62. ^{13}C-NMR spectrum of **7** in C_5D_5N at 298K (150 MHz)

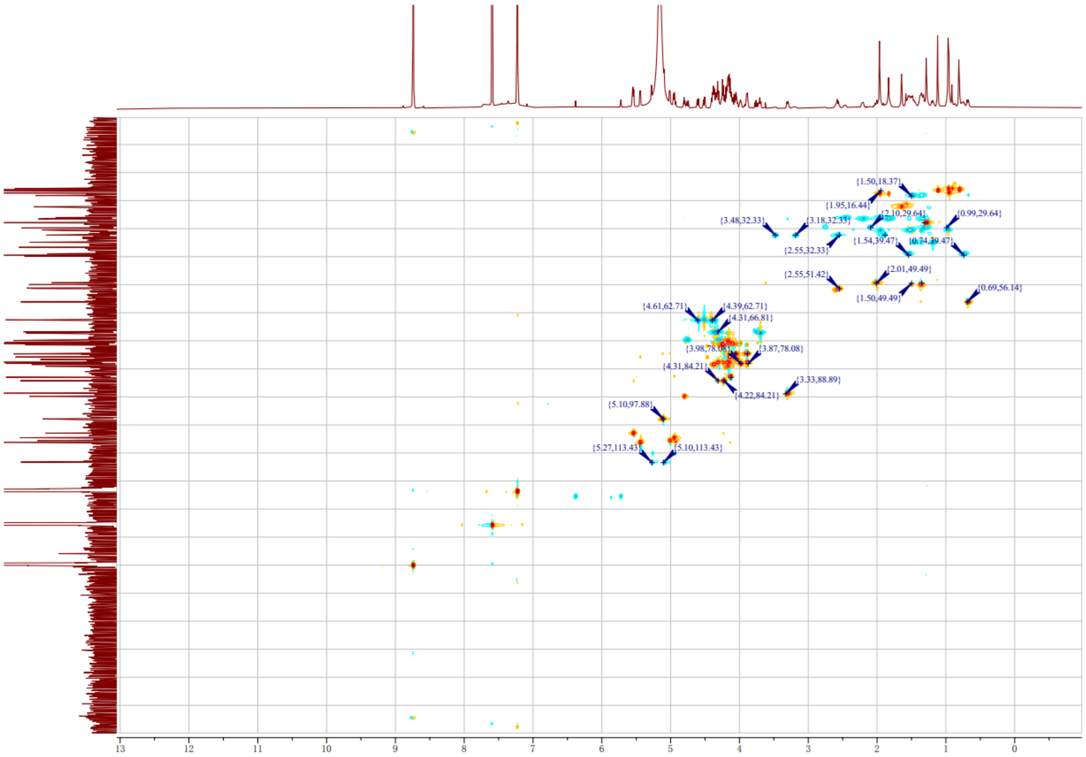

Figure S63. HSQC spectrum of **7** in C_5D_5N at 298K

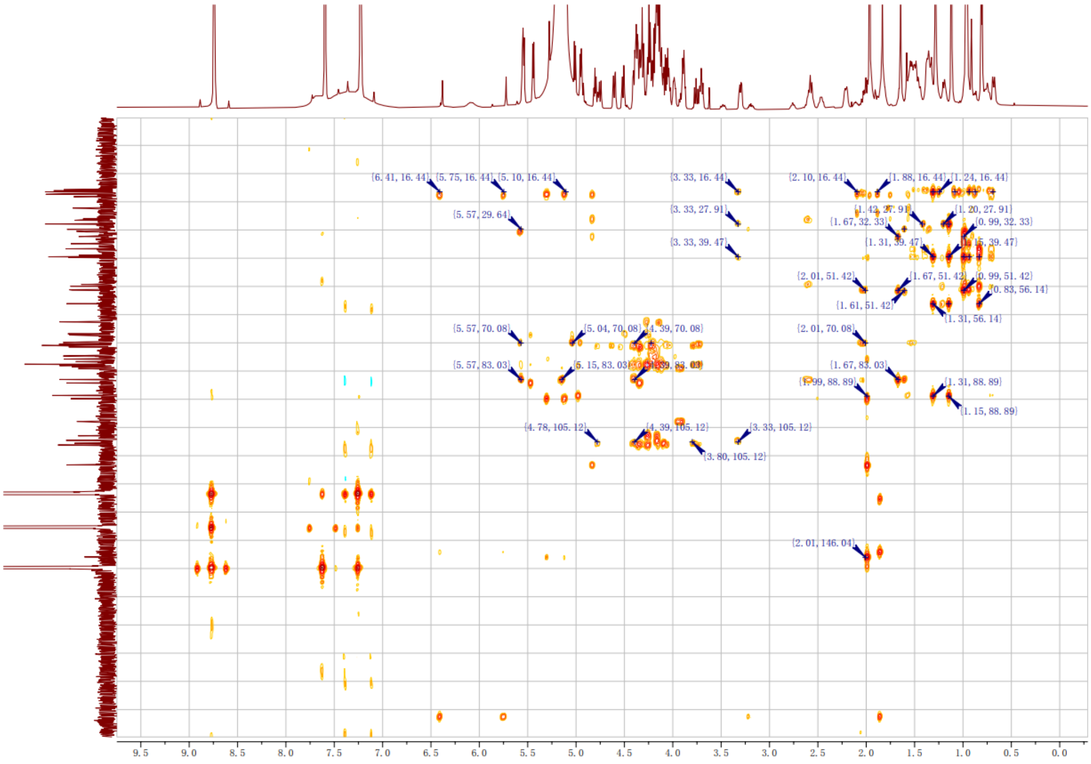

Figure S64. HMBC spectrum of **7** in C_5D_5N at 298K

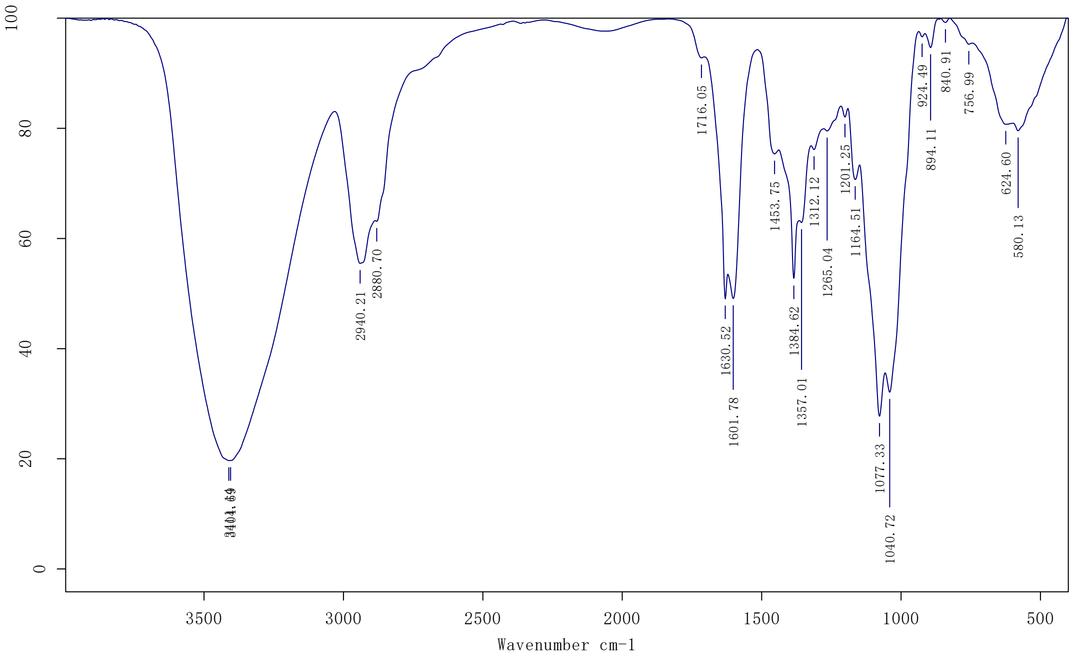

Figure S65. IR spectrum of **8**

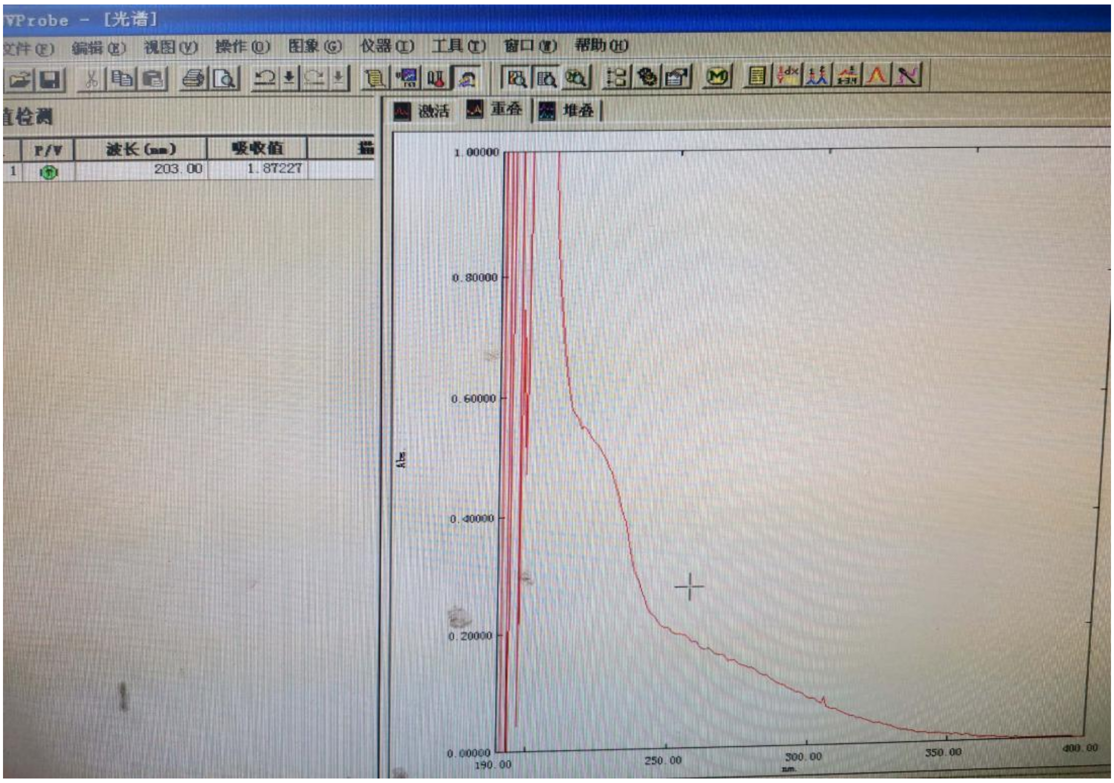

Figure S66. UV spectrum of **8**

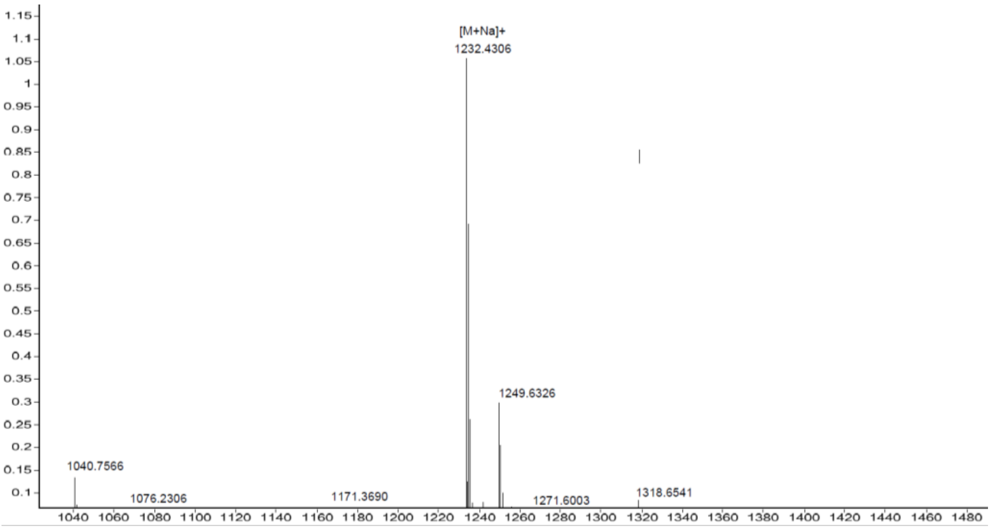

Figure S67. HRESI spectrum of **8**

Rudolph Research Analytical

This sample was measured on an Autopol VI, Serial #91058
Manufactured by Rudolph Research Analytical, Hackettstown, NJ, USA.

Measurement Date : Thursday, 27-FEB-2020

Set Temperature : OFF

Time Delay : Disabled

Delay between Measurement : Disabled

n	Average	Std.Dev.	% RSD	Maximum	Minimum
5	-7.20	0.45	-6.25	-6.50	-7.50

S.No	Sample ID	Time	Result	Scale	OR °Arc	WLG.nm	Lg.mm	Conc.g/100ml	Temp.
1	GY-29	03:01:01 PM	-7.50	SR	-0.015	589	100.00	0.200	23.6
2	GY-29	03:01:07 PM	-7.50	SR	-0.015	589	100.00	0.200	23.6
3	GY-29	03:01:13 PM	-7.50	SR	-0.015	589	100.00	0.200	23.6
4	GY-29	03:01:18 PM	-6.50	SR	-0.013	589	100.00	0.200	23.6
5	GY-29	03:01:24 PM	-7.00	SR	-0.014	589	100.00	0.200	23.6

Figure S68. $[\alpha]_D^{24}$ of **8**

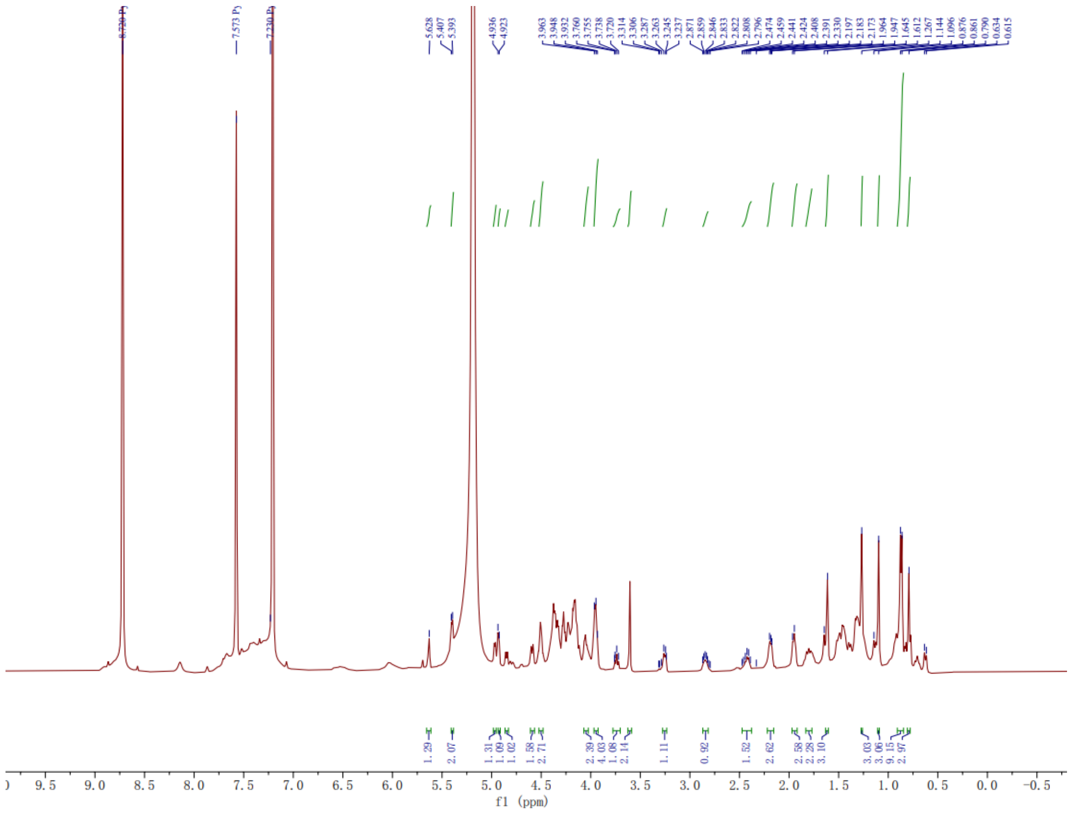

Figure S69. ^{1}H-NMR spectrum of **8** in C_5D_5N at 298K (600 MHz)

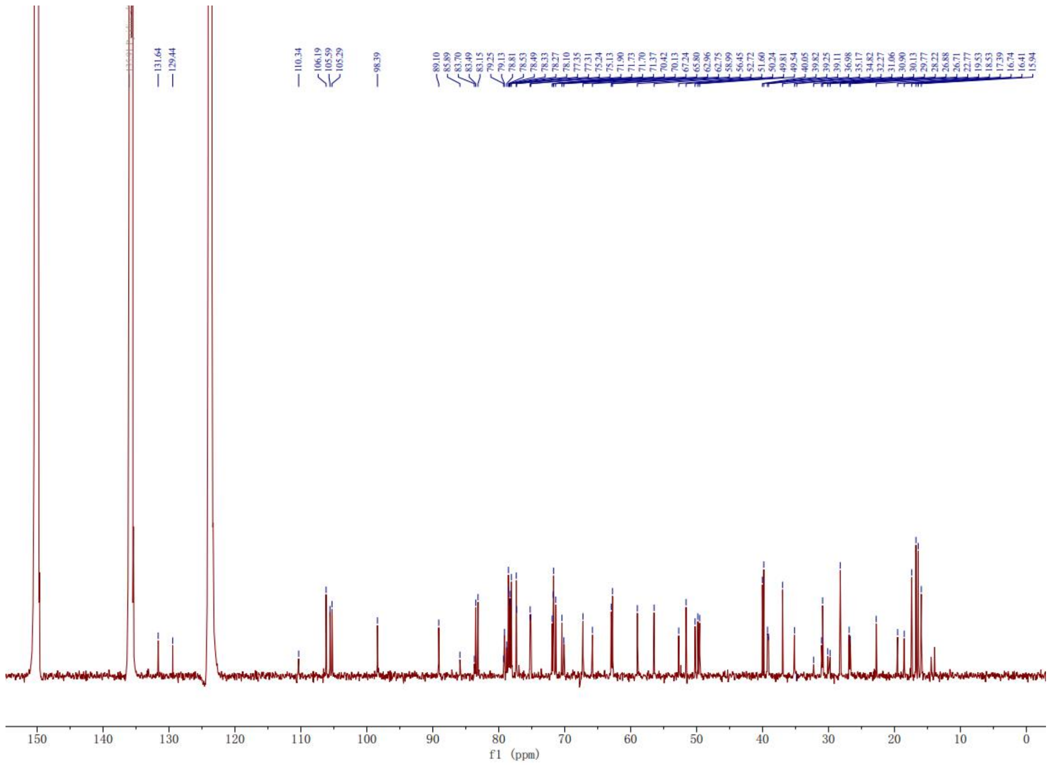

Figure S70. ^{13}C-NMR spectrum of **8** in C_5D_5N at 298K (150 MHz)

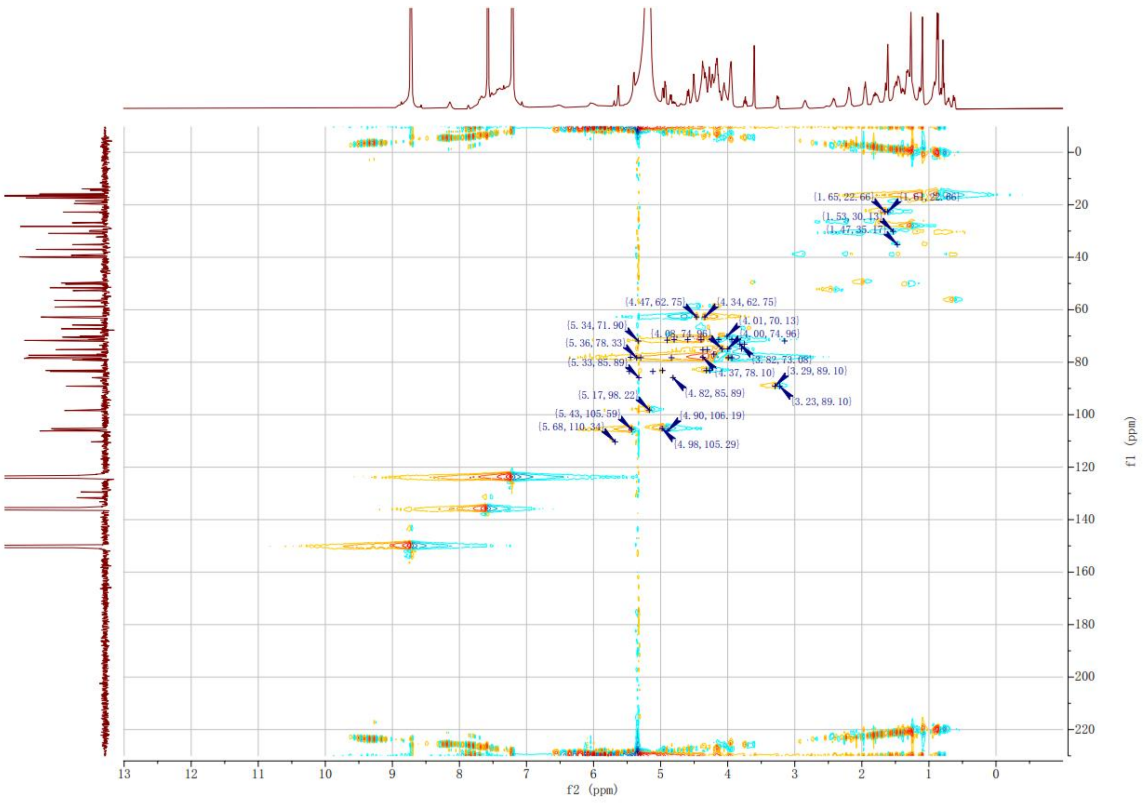

Figure. S71. HSQC spectrum of **8** in C_5D_5N at 298K

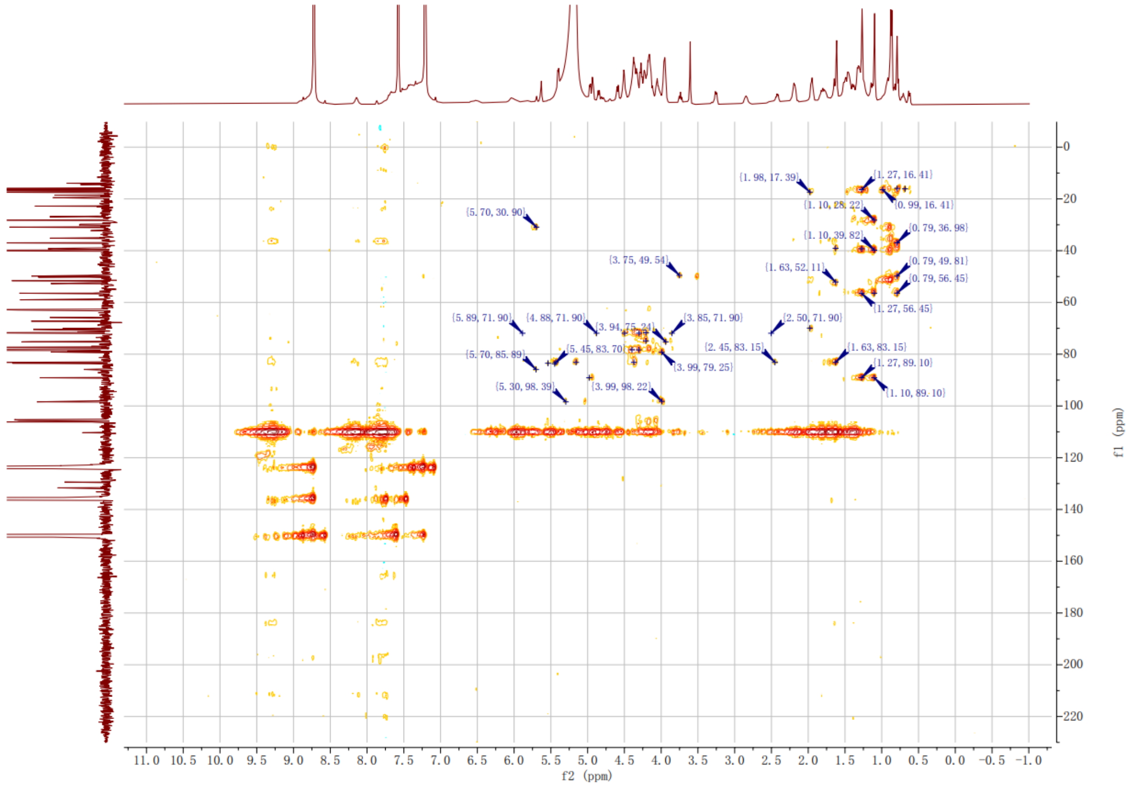

Figure S72. HMBC spectrum of **8** in C_5D_5N at 298K
